# Impact of the Autism-Associated Long Noncoding RNA *MSNP1AS* on Neuronal Architecture and Gene Expression in Human Neural Progenitor Cells

**DOI:** 10.3390/genes7100076

**Published:** 2016-09-28

**Authors:** Jessica J. DeWitt, Nicole Grepo, Brent Wilkinson, Oleg V. Evgrafov, James A. Knowles, Daniel B. Campbell

**Affiliations:** 1 Zilkha Neurogenetic Institute, Keck School of Medicine, University of Southern California, Los Angeles, CA 90089, USA; jjdewitt@usc.edu (J.J.D.); grepo@usc.edu (N.G.); brentwil@usc.edu (B.W.); evgrafov@med.usc.edu (O.V.E.); knowles@med.usc.edu (J.A.K.); 2 Department of Psychiatry and the Behavioral Sciences, Keck School of Medicine, University of Southern California, Los Angeles, CA 90089, USA

**Keywords:** lncRNA, RNA-sequencing, autism, long noncoding RNA, noncoding RNA, neuronal progenitor

## Abstract

We previously identified the long noncoding RNA (lncRNA) *MSNP1AS* (moesin pseudogene 1, antisense) as a functional element revealed by genome wide significant association with autism spectrum disorder (ASD). *MSNP1AS* expression was increased in the postmortem cerebral cortex of individuals with ASD and particularly in individuals with the ASD-associated genetic markers on chromosome 5p14.1. Here, we mimicked the overexpression of *MSNP1AS* observed in postmortem ASD cerebral cortex in human neural progenitor cell lines to determine the impact on neurite complexity and gene expression. ReNcell CX and SK-N-SH were transfected with an overexpression vector containing full-length *MSNP1AS*. Neuronal complexity was determined by the number and length of neuronal processes. Gene expression was determined by strand-specific RNA sequencing. *MSNP1AS* overexpression decreased neurite number and neurite length in both human neural progenitor cell lines. RNA sequencing revealed changes in gene expression in proteins involved in two biological processes: protein synthesis and chromatin remodeling. These data indicate that overexpression of the ASD-associated lncRNA *MSNP1AS* alters the number and length of neuronal processes. The mechanisms by which *MSNP1AS* overexpression impacts neuronal differentiation may involve protein synthesis and chromatin structure. These same biological processes are also implicated by rare mutations associated with ASD, suggesting convergent mechanisms.

## 1. Introduction

Autism spectrum disorder (ASD) is a neurodevelopmental disorder characterized by deficits in social communication and repetitive behaviors with restricted interests [1,2]. Highly penetrant, rare, de novo loss of function mutations has been associated with ASD, and many of the associated genes converge upon the biological processes of protein synthesis and chromatin structure [3,4]. However, 40% of the heritability of ASD resides in common genetic variants [5,6]. The first reported common genetic variants with genome-wide significant association with ASD mapped to a small cluster of chromosome 5p14.1, including the common genetic variant rs4307059 with ASD association of *p* = 10^−10^ [7]. The same rs4307059 allele was also identified as a predictor or stereotyped conversation and poorer communication skills in a population-based sample of >7000 individuals [8], suggesting that rs4307059 may be a quantitative trait locus for social communication phenotypes. We identified a 3.9 kb long noncoding RNA (lncRNA) that is transcribed directly at the site of the chromosome 5p14.1 ASD association signal [9]. The lncRNA is encoded by the opposite (anti-sense) strand of moesin pseudogene 1 (*MSNP1*), and is thus designated *MSNP1AS* (moesin pseudogene 1, anti-sense). Expression of *MSNP1AS* in the postmortem temporal cortex is increased 12.7-fold in individuals with ASD and increased 22-fold in individuals with the rs4307059 risk allele [9]. Thus, our discovery revealed an lncRNA, which based on the highly significant genetic association findings [7], contributes to ASD risk [9].

*MSNP1AS* is 94% identical and anti-sense to the X chromosome transcript *MSN*, which encodes a protein (moesin) that regulates neuronal architecture [10,11,12,13,14]. The lncRNA *MSNP1AS* binds *MSN* and its over-expression in cell lines caused significant decreases in moesin protein [9], which influences neuronal process stability [12]. Based on these observations, a direct hypothesis is that overexpression of *MSNP1AS* in progenitors of cortical projection neurons will decrease neuronal complexity, consistent with observations of decreased long distance connectivity observed in the cerebral cortex of individuals with ASD [15,16,17,18,19,20]. We tested this hypothesis by transfecting differentiating human neural progenitor cells with an overexpression vector that drives full-length *MSNP1AS* and directly measuring the number and length of the neurites. We demonstrated previously that one mechanism by which *MSNP1AS* may alter neuronal complexity is by decreasing the expression of moesin protein [9]. However, alternative hypotheses about the mechanism(s) of the ASD-associated lncRNA *MSNP1AS* had not been tested. While our previous experiments revealed that *MSNP1AS* over-expression decreased expression of moesin protein [9], it was not yet clear if *MSNP1AS* regulated the expression of the *MSN* transcript or if *MSNP1AS* over-expression altered the expression of other genes. Therefore, we performed unbiased RNA sequencing on differentiating human neural progenitor cells to determine the impact of increased *MSNP1AS* on gene expression. These experiments mimic the increased expression of *MSNP1AS* observed in postmortem brains of individuals with ASD and reveal multiple mechanisms by which the lncRNA *MSNP1AS* may contribute to altered neuronal architecture.

## 2. Materials and Methods

### 2.1. Cell Culture

The human neural progenitor cell lines SK-N-SH cells (American Type Culture Collection, Manassas, VA, USA) and ReNcell CX cells (Millipore, Billerica, MA, USA) were cultured according to the manufacturer’s protocols and maintained in a 75 cm^2^ flask at 37 °C and 5% CO_2_. When cells were 75% confluent, the human neural progenitor cells were subcultured to a density of 1 × 10^6^ cells per 75 cm^2^ flask for harvests 24 h post-transfection, and 5 × 10^5^ cells per 75 cm^2^ flask for harvests 72 h post-transfection.

### 2.2. Transfection of Over-Expression Constructs

Full-length *MSNP1AS* was inserted into pIRES2-AcGFP (Clontech) [9], a mammalian over-expression construct. Each experiment was made up of one pIRES2-AcGFP negative control transfection and one pIRES2-AcGFP-*MSNP1AS* transfection. Cells were transfected using Amaxa Nucleofector (Lonza, Walkersville, MD, USA) technology, using 2 µg of vector per well, and subcultured into 6-well plates. One milliliter of fresh prewarmed media was added to each well and the cells were centrifuged twice at 130× *g* for 10 min for the SK-N-SH cells and 300× *g* for 5 min for the ReNcell CX cells with PBS washes of 7 mL and 4 mL, respectively. The cell pellet was resuspended in Nucleofector solution containing a supplement using 1 × 10^6^ cells per well. One hundred microliters of cell/Nucleofector solution was added to a new cuvette and placed in the Amaxa Nucleofector using the T-16 program. Five hundred microliters of fresh prewarmed media was added to the cuvette, mixed, and transferred to the well with a disposable pipet. A two mL culture medium was added to the transfected cells in the 6-well plate. The cells were incubated at 37 °C in 5% CO_2_ until harvest. Each experiment was repeated four times.

### 2.3. Imaging

At 24 and 72 h post-transfection, human neural progenitors were viewed using an Olympus CKX41 inverted microscope and an attached Q Imaging QICAM Fast 1394 Digital Camera captured digital images. Randomly selected fields of the 6-well plate were imaged. Three randomly selected fields were imaged in each experiment. Neurite length and number of each GFP-positive cell within the field were quantified using Autoneuron software. Only neurons unobstructed by other neurons or debris were analyzed. Statistical significance was calculated using the Mann-Whitney *U* test.

### 2.4. Neural Progenitor Cell Harvest for RNA Purification

After 24 h and 72 h, the culture medium was removed and discarded. The cells were washed with one mL of PBS wash and the PBS wash was aspirated. One mL of Trypsin/EDTA solution for SK-N-SH cells and Accutase for ReNcell CX cells was added to each well and the cells were incubated for 4 min. Five mL of cell type-specific medium was added and cells were triturated and transferred to 15 mL conical tubes. SK-N-SH cells were centrifuged at 130× *g* for 10 min and ReNcell CX cells were centrifuged at 300× *g* for 5 min. The supernatant was aspirated and 5 mL of PBS added. The cell pellet was triturated and centrifuged at 130× *g* for 10 min and 300× *g* for 5 min respectively. The PBS wash was aspirated and 1 mL of fresh PBS was added. Half of the solution was transferred to each of two 1.5 mL eppendorf tubes. The tubes were centrifuged at 4 °C at 130× *g* for 10 min for SK-N-SH cells and 300× *g* for 5 min for ReNcell CX cells. The supernatant was decanted and the cell pellets were frozen at −80 °C.

### 2.5. RNA Purification

The Qiagen RNEasy kit was used to isolate total RNA using vacuum technology according to the manufacturer’s protocol (Qiagen, Valencia, CA, USA). The RNA was eluted with 35 microliters of RNAse-free water and quantified using the NanoDrop ND-1000 Spectrophotometer (v3.1.2; Thermo Fisher Scientific, Waltham, MA, USA). The RNA was stored at −20 °C.

### 2.6. Quantitative RT-PCR (qRT-PCR)

To confirm overexpression of *MSNP1AS*, consistent with the ~12-fold increase observed in postmortem brains of individuals with ASD, cDNA was synthesized using the SuperScript III First-Strand Synthesis System for qRT-PCR protocol (Invitrogen/Thermo Fisher Scientific, Waltham, MA, USA). Five hundred nanograms of RNA were used to make one and a half reaction volumes (35 μL) of cDNA. The cDNA was stored at −20 °C. The qRT-PCR protocol described in Kerin et al. [9] was used to validate over-expression of *MSNP1AS*.

### 2.7. Construction of Strand-Specific, Ribosomal RNA Depleted RNA Sequencing Libraries

Directional RNA-Seq libraries were prepared for Illumina HiSeq 2000 sequencing using the Stranded Total TruSeq RNA Sample Preparation kit with Ribo-Zero Gold (Illumina) using the manufacturer’s protocol using the Hamilton Starlet Liquid Handling robot. One nanogram of RNA was used for each sample. In brief, Ribo-Zero was used to deplete cytoplasmic and mitochondrial rRNA from total RNA. The depleted RNA was fragmented and primed with random hexamers to synthesize first strand cDNA using Superscript II (Life Technologies, Carlsbad, CA, USA). Next, the second strand was synthesized, incorporating dUTP in place of dTTP. A single “A” base was added to the 3′ ends of the fragments and the indexed adaptors were ligated to the ends of the ds cDNA to prepare them for hybridization onto the flow cell. PCR was used to selectively enrich the fragments with ligated adapters and to amplify the amount of DNA in the library. The libraries were produced in a 96-well format and quality controlled using the Agilent Technologies 2200 TapeStation Instrument. Libraries were pooled (four samples per lane) and sequenced on Illumina HiSeq 2000 to a targeted depth, generating an average of 20 million paired-end 50-cycle reads for each sample (Table S1).

### 2.8. Data Analysis

Data analysis was performed using TopHat (version 2.0.10; https://ccb.jhu.edu/software/tophat/index.shtml) [21] to align the Illumina short reads against the reference human genome ENSEMBL GrCH38 version 81. Sequence alignments were generated as BAM files [22], and then Cuffdiff (version 2.2.1; http://cole-trapnell-lab.github.io/cufflinks/) [23] was used to summarize the gene expression values as FPKM measures. The gene expression of samples with *MSNP1AS* over-expression were compared to the gene expression of the negative control experiment samples to find other differentially-expressed genes. Cuffdiff was also used to calculate the expression fold change, *p*-values and FDR values. Genes with *p* < 0.05 were used as the input for DAVID (version 6.7; Leidos Biomedical Research, Inc., Frederick, MD, USA) functional annotation [24,25].

## 3. Results

### 3.1. Overexpression of MSNP1AS Decreased Neurite Number and Length in SK-N-SH and ReNcell CX Human Neural Progenitor Cells

**Figure 1:**
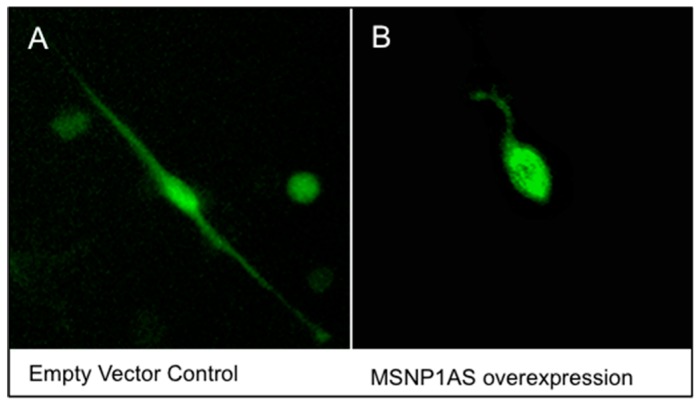
Representative ReNcell CX cells at 24 h post-transfection. The human neural progenitor cells were transfected with (**A**) an empty vector control or (**B**) the *MSNP1AS* overexpression vector. For each experiment, the transfection of *MSNP1AS* over-expression or control vector was repeated four times. Three randomly selected fields from each of the four replicates were imaged and all isolated GFP-positive cells within the imaged fields were examined for neurite length and neurite number using Autoneuron software.

**Figure 2:**
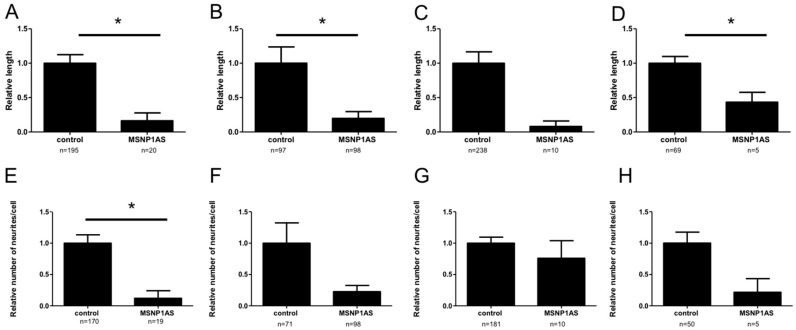
Overexpression of *MSNP1AS* decreases neurite number and length. SK-N-SH cells that overexpressed *MSNP1AS* had decreased neurite length after (**A**) 24 h and (**B**) 72 h. ReNcell CX cells that overexpressed *MSNP1AS* also had decreased neurite length after (**D**) 72 h but the decreased neurite length after (**C**) 24 h was not significant. SK-N-SH cells that overexpressed *MSNP1AS* had fewer neurites per cell after (**E**) 24 h and a trend toward reduced neurites per cell after (**F**) 72 h. ReNcell CX cells that overexpressed *MSNP1AS* also had trend toward fewer neurites per cell after (**H**) 72 h, but the neurite number decrease after (**G**) 24 h was not significant (* *p* < 0.05, Mann-Whitney *U* test).

To mimic the overexpression of *MSNP1AS* observed in postmortem brains of individuals with ASD, *MSNP1AS* was overexpressed ~50-fold (Table S2) in human neural progenitor cells using a human overexpression vector containing the full length *MSNP1AS* transcript. Human neural progenitor cells that overexpressed *MSNP1AS* had decreased neurite length compared to human neural progenitor cells transfected with the empty vector control (Figure 1 and Figure 2). In SK-N-SH cells, neurite length was reduced 6-fold (*p* = 1.1 × 10^−2^) at 24 h post-transfection and 5-fold (*p* = 6.0 × 10^−4^) at 72 h post-transfection in neural progenitor cells transfected with the *MSNP1AS* over-expression vector compared to controls (Figure 2A,B). Similarly, *MSNP1AS* overexpression in ReNcell CX human neural progenitor cells caused a significant decrease in neurite length at 72 h post-transfection by 12.5-fold (*p* = 2.5 × 10^−2^), but the decreased neurite length observed at 24 h post-transfection was not significant (2.3-fold; *p* = 2.9 × 10^−1^) (Figure 2C,D).

*MSNP1AS* overexpression also caused a reduction in the number of neurites in human neural progenitor cells. In SK-N-SH cells, *MSNP1AS* overexpression caused an 8.3-fold reduction in neurite number at 24 h post-transfection (*p* = 2.1 × 10^−2^) and a trend toward reduced neurite number at 72 h post-transfection (4.4-fold; *p* = 6.2 × 10^−2^) (Figure 2E,F). Similarly, *MSNP1AS* overexpression in ReNcell CX cells caused a trend toward fewer neurite number at 72 h post-transfection (4.6-fold; *p* = 1.0 × 10^−1^), but the neurite number decrease at 24 h post-transfection was not significant (1.3-fold; *p* = 7.9 × 10^−1^) (Figure 2G,H).

### 3.2. Genome-Wide Changes in Gene Expression Following MSNP1AS Overexpression in Human Neural Progenitor Cells

Changes in the transcriptome caused by *MSNP1AS* overexpression may provide insight into the mechanisms by which an increase of *MSNP1AS* influences neuronal architecture. Therefore, RNA sequencing was used to perform genome-wide transcriptome profiling on the two human neural progenitor cells lines SK-N-SH and ReNcell CX cells with and without *MSNP1AS* overexpression. For all experiments, the average number of reads ranged from 14–29 million with 96% of the reads mapping to the genome (Table S1). No change in gene expression survived Bonferroni correction for multiple comparisons in any of the experiments (Table S3). However, each of the experiments revealed 100–400 genes with nominally significant changes in gene expression (*p* < 0.05). All downstream analyses were performed with the sets of genes that had significant (*p* < 0.05) changes in expression following *MSNP1AS* over-expression. Differential gene expression analysis in SK-N-SH cells revealed 157 differentially expressed genes (*p* < 0.05) at 24 h post-transfection and 351 genes differentially expressed (*p* < 0.05) at 72 h post-transfection (Table S3). In ReNcell CX cells, *MSNP1AS* overexpression revealed 267 genes differentially expressed at 24 h post-transfection and 164 genes differentially expressed at 72 h post-transfection (Table S3). No single gene was significantly (*p* < 0.05) altered in each of the four experimental conditions (Table S3).

*MSNP1AS* overexpression did not alter expression of *MSN*, the transcript that encodes moesin protein and is bound by the *MSNP1AS* lncRNA. In SK-N-SH cells, *MSN* transcript was slightly increased at 24 h post-transfection (1.17-fold; *p* = 3.0 × 10^−2^) and at 72 h post-transfection (1.16-fold; *p* = 3.0 × 10^−1^). In ReNcell CX cells, a significant change in *MSN* transcript expression was not observed at 24 h post-transfection (0.97-fold; *p* = 8.0 × 10^−1^) and at 72 h post-transfection (0.92-fold; *p* = 5.0 × 10^−1^). These results suggest that, although *MSNP1AS* regulates expression of moesin protein [9], *MSNP1AS* does not directly regulate the expression of the *MSN* transcript.

### 3.3. Transcriptional Consequences of MSNP1AS Overexpression Are Enriched in Protein Synthesis and Chromatin Regulation

**Figure 3:**
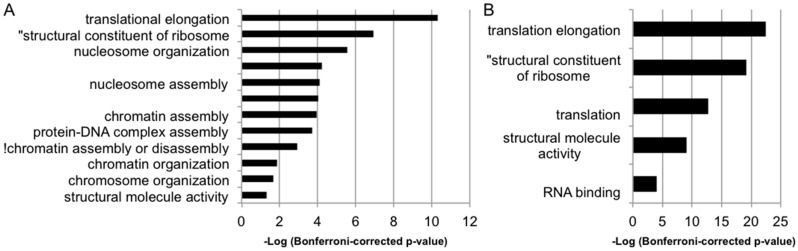
Gene Ontology (GO) enrichment analysis on all differentially expressed genes due to overexpression of *MSNP1AS*. GO enrichment analysis was performed on (**A**) SK-N-SH cells after 72 h, and (**B**) ReNcell CX cells after 24 h.

Gene ontology (GO) analysis using the Database for Annotation, Visualization and Integrated Discovery (DAVID) web server for differentially expressed (*p* < 0.05) genes revealed an enrichment of genes involved in protein synthesis and chromatin regulation (Figure 3A; Table S4). In SK-N-SH cells at 72 h post-transfection of the *MSNP1AS* overexpression construct, the 351 genes with altered expression (*p* < 0.05) were enriched for genes involved in translational elongation (Bonferroni corrected *p* = 4.7 × 10^−11^), structural constituent of ribosome (Bonferroni corrected *p* = 1.2 × 10^−7^), and nucleosome organization (Bonferroni corrected *p* = 2.8 × 10^−6^). No enrichment that survived Bonferroni correction was observed among the 157 differentially expressed genes in SK-N-SH cells at 24 h post-transfection. However, a trend toward enrichment of chromatin related genes was observed in SK-N-SH cells at 24 h (uncorrected *p* = 5.8 × 10^−2^) with some of the same genes differentially expressed as SK-N-SH at 72 h post-transfection (e.g., *HIST1H2AJ*, Table S4).

Similarly, in ReNcell CX human neural progenitor cells at 24 h post-transfection, differentially expressed (*p* < 0.05) genes were enriched for translational elongation (Bonferroni corrected *p* = 3.3 × 10^−23^), structural constituent of ribosome (Bonferroni corrected *p* = 7.3 × 10^−20^), and translation (Bonferroni corrected *p* = 1.7 × 10^−13^) (Figure 3B; Table S4). No enrichment that survived Bonferroni correction was observed among the 164 genes differentially expressed in ReNcell CX cells at 72 h post-transfection. However, an enrichment of genes involved in translation (uncorrected *p* = 4.0 × 10^−2^) was among the top results in ReNcell CX cells at 72 h post-transfection. These data suggest that genes involved in translation are altered by *MSNP1AS* overexpression.

## 4. Discussion

**Figure 4:**
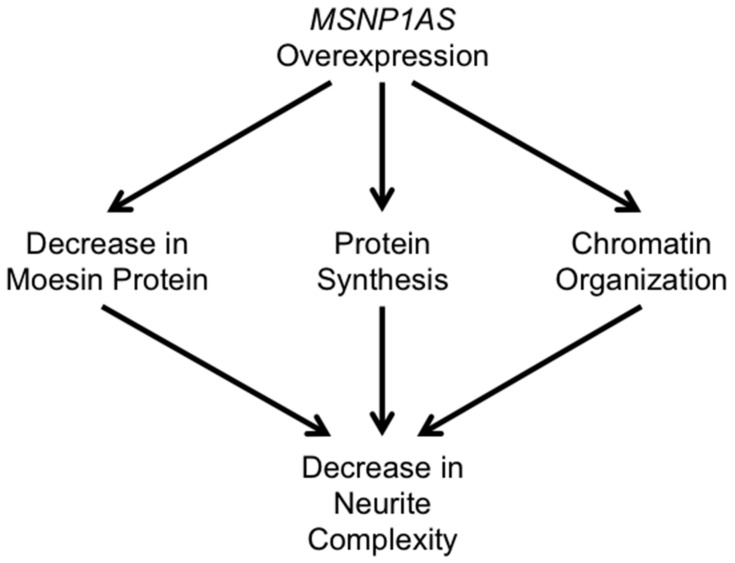
Potential mechanisms by which *MSNP1AS* alters neuronal architecture. *MSNP1AS* overexpression decreases the expression of moesin protein, as well as the expression of genes involved in protein synthesis and chromatin organization, leading to a decrease in neurite complexity.

The data presented here indicate that overexpression of the ASD-associated lncRNA *MSNP1AS* alters neuronal architecture in human neural progenitor cells by three potential mechanisms (Figure 4). As we previously described [9], *MSNP1AS* overexpression decreases expression of moesin protein, which is known to decrease neuronal complexity [12]. Here, we demonstrate that *MSNP1AS* overexpression also changes the expression of genes involved in protein synthesis and chromatin organization. Therefore, *MSNP1AS* overexpression has multiple molecular functions: *MSNP1AS* alters expression of genes that contribute to chromatin organization; *MSNP1AS* binds *MSN* and alters the translation of moesin protein; and *MSNP1AS* alters the expression of genes that regulate translation more globally (Figure 4).

*MSNP1AS* was discovered as the functional element revealed by an ASD genome-wide association study [9]. The allele frequencies of the chromosome 5p14.1 genetic variants with genome-wide significant association are high: greater than half the population carries at least one copy of the risk allele [7]. It is unclear whether individuals who are homozygous for the rs4307059 risk allele are at higher risk for ASD than those heterozygous at rs4307059. However, the expression of *MSNP1AS* is increased in the postmortem temporal cortex of individuals who are homozygous for rs4307059 compared to individuals who are heterozygous for rs4307059 [9]. It is striking that the biological functions of this ASD-associated lncRNA—protein synthesis and chromatin organization—are matched closely to the biological functions revealed by genes with rare de novo mutations associated with ASD [26,27]. These results suggest that both common and rare ASD-associated variants converge upon the common molecular pathways. Further, the chromosome 5p14.1 genetic marker with the most significant association (rs4307059 with ASD association *p* = 10^−10^) also contributes to altered social communication in a general population sample [8]. Together, these data suggest common molecular pathways that contribute to social communication that involve protein translation and chromatin organization.

*MSNP1AS* is antisense to moesin (membrane-organizing extension spike protein). Moesin is a member of the ERM (exrin/radixin/moesin) family of proteins that link the actin filaments to the cellular membrane. Along with radixin, moesin protein localizes to growth cones and filopodia that emanate from neurite shafts, both regions of high motility and growth. These proteins are especially important during development, as shown in rat cerebral cortex. In rats, ERM protein expression reaches a maximum near birth and gradually declines through postnatal development [12], which is during synaptic maturation. In the intermediate zone of developing rat cerebral cortex, ERM proteins are significantly expressed in neurite extensions at embryonic day 17 [11], a time of substantial synapse formation. They regulate adhesion receptors, signaling molecules that provide spatial information to the cell, and growth cone actin to mediate attractive growth cone guidance [28]. When antisense RNA is used to knockdown moesin in cultured hippocampal and cortical neurons, specific phenotypes are observed, including a dramatic reduction in the rate of neurite advancement [12], suppression of neurite formation [10], growth cone collapse [12], suppression of an increase in dendritic spine formation induced by estrogen [14], and suppression of an increase in active presynaptic boutons induced by glutamate [13]. In addition, ERM proteins have been shown to mediate neuritogenesis [29]. These data denote that the function of moesin involves both regulating axonal growth cone development presynaptically and initiating dendritic spine development postsynaptically. Moesin knockout mice have not been evaluated for behavioral phenotypes or brain development.

While *MSN* transcripts did not undergo a significant change in gene expression, significant alterations are observed in neurite development. As we previously described, overexpressed *MSNP1AS* binds *MSN* transcript and prevents translation of moesin protein. The current study expands our knowledge of *MSNP1AS* overexpression in two ways. First, the observation that *MSNP1AS* overexpression does not alter expression of *MSN* transcript indicates that *MSNP1AS* does not directly contribute to the regulation of the *MSN* transcription. Instead, in relation to the regulation of moesin, it appears that *MSNP1AS* acts only to inhibit the translation of moesin protein. Second, *MSNP1AS* acts more globally than just inhibiting the translation of moesin. The overexpression of *MSNP1AS* caused changes in the expression of genes involved in the protein synthesis and chromatin regulation. These transcriptional changes suggest that *MSNP1AS* participates in global biological processes that impact neuronal differentiation beyond its impact on moesin. Additional experiments will be necessary to determine the relative contributions of *MSNP1AS* to the biological processes of chromatin regulation, protein synthesis, and the regulation of moesin. It will be important to determine the expression of moesin protein and the *MSNP1AS* noncoding RNA in neurons derived from patients with the ASD-associated rs4307059 allele, as well as from patients with Cri-du-chat syndrome with deletion of chromosome 5p14.1, as these experiments may provide further evidence of a contribution of *MSNP1AS* to altered brain development. It will also be important to determine if individuals with ASD and the ASD-associated rs4307059 allele exhibit common comorbid features of ASD, such as gastrointestinal disorders or epilepsy. A network analysis of microarray data from postmortem ASD brain placed *MSN* as a central node [30]. However, there was no enrichment of the genes in the *MSN* node and the genes with altered expression following *MSNP1AS* over-expression identified here.

In this study, overexpressed *MSNP1AS* results in shortened neurites and fewer neurites per cell, indicating that neuritogenesis and extension are both impacted. The decreased neurite number and growth upon overexpression of *MSNP1AS* reinforces studies that suggest that altered short- and long-range connectivity in the brains of autism patients may be contributing to the pathogenesis of the disorder [31]. This is in line with global observations of decreased connectivity in the brains of individuals with ASD [16,17,18,19,20,32], although there is evidence for increased connectivity between some brain regions in ASD [19,20].

Noncoding RNA comprise 90% of the human genome and are believed to contribute to regulatory function as enhancers and promoters [33,34]. These elements differentiate across space and time in a significant way, but the exact functional roles of most lncRNAs have not been quantified [35,36,37]. The contributions of lncRNAs to psychiatric disorders are a topic of ongoing research [38]. The data from this study provide further evidence for pathways reported to influence ASD, as well as giving additional insight into the molecular function of the ASD-associated lncRNA, *MSNP1AS*.

## 5. Conclusions

We previously identified the lncRNA *MSNP1AS* as a functional element revealed by genome wide significant association with ASD. Using a candidate gene approach, we showed that one function of *MSNP1AS* is to regulate the expression of moesin protein. The data presented here indicate that *MSNP1AS* over-expression does not significantly alter the expression of the *MSN* transcript, suggesting that *MSNP1AS* functions specifically at regulating the translation of the *MSN* transcript to moesin protein. Further, the unbiased RNA sequencing data revealed that over-expression of *MSNP1AS* alters the expression of genes involved in protein synthesis and chromatin organization. These biological processes are also implicated by rare mutations associated with ASD, suggesting convergent molecular mechanisms that contribute to the altered brain development of ASD.

## Supplementary Materials

The following are available online at www.mdpi.com/2073-4425/7/10/76/s1, Table S1: RNA sequencing metrics, Table S2: quantitative over-expression of *MSNP1AS*, Table S3: genes differentially expressed following *MSNP1AS* over-expression, Table S4: gene networks differentially expressed following *MSNP1AS* over-expression.
