# Supplementary material for: Impact of the Autism-Associated Long Noncoding RNA MSNP1AS on Neuronal Architecture and Gene Expression in Human Neural Progenitor Cells
Source: Genes (Basel). 2016 Sep 28;7(10):76. doi: 10.3390/genes7100076 (PMC5083915; doi:10.3390/genes7100076)
Supplement: Supplementary file 1 [file genes-07-00076-s001.docx]

Supplementary Materials: Impact of the Autism−Associated Long Noncoding RNA *MSNP1AS* on Neuronal Architecture and Gene Expression in Human Neural Progenitor Cells

Jessica J. DeWitt, Nicole Grepo, Brent Wilkinson, Oleg V. Evgrafov, James A. Knowles, Daniel B. Campbell

**Table S1**. RNA Sequencing Metrics

|  | **Number of reads** | **Number of reads mapped** | **% mapped** |
| --- | --- | --- | --- |
| **SK-N-SH 24 h** |  |  |  |
| control biological replicate 1 | 11,099,318 | 10,746,390 | 96.8 |
| control biological replicate 2 | 20,496,459 | 19,422,292 | 94.8 |
| control biological replicate 3 | 10,824,013 | 10,458,919 | 96.6 |
| control biological replicate 4 | 31,400,592 | 29,709,967 | 94.6 |
| Average | 18,455,096 | 17,584,392 | 95.7 |
| MSNP1AS OE biological replicate 1 | 19,971,956 | 19,337,134 | 96.8 |
| MSNP1AS OE biological replicate 2 | 13,807,707 | 12,881,017 | 93.3 |
| MSNP1AS OE biological replicate 3 | 18,326,170 | 17,660,733 | 96.4 |
| MSNP1AS OE biological replicate 4 | 10,900,335 | 10,366,157 | 95.1 |
| Average | 15,751,542 | 15,061,260 | 95.4 |
| **SK-N-SH 72 h** |  |  |  |
| control biological replicate 1 | 46,378,868 | 44,126,735 | 95.1 |
| control biological replicate 2 | 22,219,398 | 20,950,969 | 94.3 |
| control biological replicate 3 | 25,049,815 | 24,123,433 | 96.3 |
| control biological replicate 4 | 24,021,135 | 23,272,061 | 96.9 |
| Average | 29,417,304 | 28,118,300 | 95.7 |
| MSNP1AS OE biological replicate 1 | 17,844,865 | 17,128,866 | 96.0 |
| MSNP1AS OE biological replicate 2 | 10,176,928 | 9,651,721 | 94.8 |
| MSNP1AS OE biological replicate 3 | 14,363,510 | 13,878,004 | 96.6 |
| MSNP1AS OE biological replicate 4 | 15,219,358 | 14,512,024 | 95.4 |
| Average | 14,401,165 | 13,792,654 | 95.7 |
| **ReNcell CX 24 h** |  |  |  |
| control biological replicate 1 | 27,003,948 | 26,267,471 | 97.3 |
| control biological replicate 2 | 20,860,192 | 19,778,456 | 94.8 |
| control biological replicate 3 | 15,548,803 | 14,713,657 | 94.6 |
| control biological replicate 4 | 26,490,502 | 25,268,925 | 95.4 |
| Average | 22,475,861 | 21,507,127 | 95.5 |
| MSNP1AS OE biological replicate 1 | 22,807,209 | 22,258,018 | 97.6 |
| MSNP1AS OE biological replicate 2 | 17,922,225 | 17,200,036 | 96.0 |
| MSNP1AS OE biological replicate 3 | 13,290,230 | 12,659,451 | 95.3 |
| MSNP1AS OE biological replicate 4 | 16,948,688 | 16,375,844 | 96.6 |
| Average | 17,742,088 | 17,123,337 | 96.4 |
| **ReNcell CX 72 h** |  |  |  |
| control biological replicate 1 | 20,666,642 | 20,014,343 | 96.8 |
| control biological replicate 2 | 14,360,503 | 13,888,459 | 96.7 |
| control biological replicate 3 | 33,262,563 | 31,348,111 | 94.2 |
| control biological replicate 4 | 35,502,944 | 34,507,246 | 97.2 |
| Average | 25,948,163 | 24,939,540 | 96.2 |
| MSNP1AS OE biological replicate 1 | 11,987,370 | 11,670,193 | 97.4 |
| MSNP1AS OE biological replicate 2 | 12,533,511 | 11,987,081 | 95.6 |
| MSNP1AS OE biological replicate 3 | 25,523,571 | 24,097,726 | 94.4 |
| MSNP1AS OE biological replicate 4 | 22,607,983 | 21,685,434 | 95.9 |
| Average | 18,163,109 | 17,360,109 | 95.8 |

**Table S2.** Quantitative Over−Expression of MSNP1AS.

| **ReNcell CX 24 h harvest** | **−ΔΔCT *MSP1AS* Over Expression** |
| --- | --- |
| biological replicate 1 | 4.95 |
| biological replicate 2 | 4.68 |
| biological replicate 3 | 2.37 |
| biological replicate 4 | 7.19 |
| Average | 4.80 |
| **ReNcell CX 72 h harvest** |  |
| biological replicate 1 | 2.87 |
| biological replicate 2 | 4.86 |
| biological replicate 3 | 5.22 |
| biological replicate 4 | 5.30 |
| Avearge | 4.56 |
| **SK-N-SH 24 h harvest** |  |
| biological replicate 1 | 12.25 |
| biological replicate 2 | 10.63 |
| biological replicate 3 | 6.45 |
| biological replicate 4 | 10.64 |
| Average | 9.99 |
| **SK-N-SH 72 h harvest** |  |
| biological replicate 1 | 9.67 |
| biological replicate 2 | 10.19 |
| biological replicate 3 | 12.61 |
| biological replicate 4 | 7.15 |
| Average | 9.91 |

**Table S3.** Genes Differentially Expressed Following MSNP1AS Over−Expression.

| **SK-N-SH 24 h** | |  |  |  |  |
| --- | --- | --- | --- | --- | --- |
| **Gene ID** | **Gene** | **Value_1** | **Value_2** | **log2 (Fold Change)** | ***p* value** |
| ENSG00000169429 | CXCL8 | 185.84 | 51.44 | −1.85 | 5.00E−05 |
| ENSG00000205336 | ADGRG1 | 1.60 | 3.39 | 1.08 | 5.00E−05 |
| ENSG00000265972 | TXNIP | 25.88 | 62.40 | 1.27 | 5.00E−05 |
| ENSG00000023445 | BIRC3 | 4.46 | 11.19 | 1.33 | 5.00E−05 |
| ENSG00000168348 | INSM2 | 3.31 | 8.80 | 1.41 | 5.00E−05 |
| ENSG00000208037 | MIR320A | 9.60 | 202.16 | 4.40 | 5.00E−05 |
| ENSG00000118785 | SPP1 | 3.63 | 0.65 | −2.48 | 0.0001 |
| ENSG00000100604 | CHGA | 29.52 | 61.47 | 1.06 | 0.00015 |
| ENSG00000134363 | FST | 162.43 | 62.68 | −1.37 | 0.0003 |
| ENSG00000087074 | PPP1R15A | 10.06 | 19.51 | 0.96 | 0.00045 |
| ENSG00000236830 | CBR3−AS1 | 2.25 | 0.68 | −1.74 | 0.0005 |
| ENSG00000105825 | TFPI2 | 86.86 | 45.43 | −0.93 | 0.00055 |
| ENSG00000133636 | NTS | 89.97 | 47.51 | −0.92 | 0.00065 |
| ENSG00000122585 | NPY | 110.99 | 64.30 | −0.79 | 0.0008 |
| ENSG00000099860 | GADD45B | 2.33 | 6.23 | 1.42 | 0.00085 |
| ENSG00000249992 | TMEM158 | 60.25 | 34.94 | −0.79 | 0.0009 |
| ENSG00000115665 | SLC5A7 | 1.50 | 3.28 | 1.13 | 0.00095 |
| ENSG00000056558 | TRAF1 | 0.89 | 2.24 | 1.34 | 0.0013 |
| ENSG00000162772 | ATF3 | 4.26 | 11.17 | 1.39 | 0.0013 |
| ENSG00000155090 | KLF10 | 33.31 | 54.20 | 0.70 | 0.00155 |
| ENSG00000116329 | OPRD1 | 0.93 | 1.84 | 0.98 | 0.00225 |
| ENSG00000159167 | STC1 | 2.88 | 1.03 | −1.48 | 0.0024 |
| ENSG00000140450 | ARRDC4 | 2.20 | 4.15 | 0.92 | 0.0024 |
| ENSG00000120738 | EGR1 | 4.57 | 8.18 | 0.84 | 0.00285 |
| ENSG00000167601 | AXL | 14.36 | 23.01 | 0.68 | 0.0029 |
| ENSG00000182109 | RP11−69E11.4 | 0.57 | 3.36 | 2.57 | 0.00295 |
| ENSG00000130707 | ASS1 | 4.74 | 1.65 | −1.52 | 0.00315 |
| ENSG00000109193 | SULT1E1 | 4.35 | 9.87 | 1.18 | 0.0033 |
| ENSG00000116717 | GADD45A | 33.31 | 53.98 | 0.70 | 0.00395 |
| ENSG00000197635 | DPP4 | 20.94 | 11.23 | −0.90 | 0.0047 |
| ENSG00000137558 | PI15 | 15.20 | 9.04 | −0.75 | 0.00505 |
| ENSG00000196954 | CASP4 | 17.43 | 9.10 | −0.94 | 0.0056 |
| ENSG00000127920 | GNG11 | 53.12 | 35.12 | −0.60 | 0.0059 |
| ENSG00000108691 | CCL2 | 127.99 | 79.50 | −0.69 | 0.00595 |
| ENSG00000101825 | MXRA5 | 5.20 | 3.21 | −0.69 | 0.00665 |
| ENSG00000204291 | COL15A1 | 10.12 | 5.78 | −0.81 | 0.0067 |
| ENSG00000125144 | MT1G | 2.14 | 0.19 | −3.46 | 0.0068 |
| ENSG00000186523 | FAM86B1 | 1.25 | 2.32 | 0.89 | 0.007 |
| ENSG00000166825 | ANPEP | 10.75 | 4.39 | −1.29 | 0.00725 |
| ENSG00000091073 | DTX2 | 1.33 | 2.93 | 1.14 | 0.0073 |
| ENSG00000174672 | BRSK2 | 1.12 | 2.90 | 1.37 | 0.0073 |
| ENSG00000123689 | G0S2 | 14.69 | 7.62 | −0.95 | 0.00755 |
| ENSG00000276644 | DACH1 | 1.22 | 2.37 | 0.96 | 0.00815 |
| ENSG00000188042 | ARL4C | 10.74 | 6.96 | −0.63 | 0.00955 |
| ENSG00000183172 | SMDT1 | 1.96 | 4.96 | 1.34 | 0.0101 |
| ENSG00000104490 | NCALD | 4.74 | 7.68 | 0.69 | 0.01045 |
| ENSG00000105894 | PTN | 73.42 | 49.00 | −0.58 | 0.0106 |
| ENSG00000162496 | DHRS3 | 1.08 | 2.43 | 1.17 | 0.01095 |
| ENSG00000111057 | KRT18 | 15.30 | 24.74 | 0.69 | 0.01105 |
| ENSG00000164434 | FABP7 | 6.68 | 3.12 | −1.10 | 0.0111 |
| ENSG00000181026 | AEN | 14.50 | 21.75 | 0.59 | 0.0111 |
| ENSG00000099194 | SCD | 42.31 | 63.41 | 0.58 | 0.01145 |
| ENSG00000139209 | SLC38A4 | 14.69 | 24.63 | 0.75 | 0.0119 |
| ENSG00000090006 | LTBP4 | 2.40 | 4.23 | 0.82 | 0.012 |
| ENSG00000100292 | HMOX1 | 2.62 | 5.21 | 0.99 | 0.01225 |
| ENSG00000277027 | RMRP | 2890.63 | 5379.38 | 0.90 | 0.013 |
| ENSG00000230461 | PROX1−AS1 | 1.17 | 2.00 | 0.77 | 0.0136 |
| ENSG00000278705 | HIST1H4B | 39.66 | 64.12 | 0.69 | 0.01385 |
| ENSG00000164761 | TNFRSF11B | 13.53 | 7.15 | −0.92 | 0.01415 |
| ENSG00000151012 | SLC7A11 | 4.94 | 7.80 | 0.66 | 0.01465 |
| ENSG00000169908 | TM4SF1 | 68.42 | 45.18 | −0.60 | 0.01505 |
| ENSG00000222328 | RNU2−2P | 568.14 | 1066.10 | 0.91 | 0.01555 |
| ENSG00000144452 | ABCA12 | 3.09 | 5.01 | 0.70 | 0.0157 |
| ENSG00000234335 | RPS4XP11 | 3.48 | 1.36 | −1.36 | 0.0158 |
| ENSG00000204915 | RP6−99M1.1 | 13.87 | 7.91 | −0.81 | 0.0164 |
| ENSG00000149948 | HMGA2 | 25.36 | 17.04 | −0.57 | 0.0172 |
| ENSG00000198899 | MT−ATP6 | 104.72 | 65.88 | −0.67 | 0.01755 |
| ENSG00000277209 | RPPH1 | 1432.18 | 2312.76 | 0.69 | 0.0178 |
| ENSG00000118507 | AKAP7 | 2.25 | 1.06 | −1.09 | 0.01785 |
| ENSG00000065268 | WDR18 | 1.94 | 4.25 | 1.13 | 0.01805 |
| ENSG00000135047 | CTSL | 41.63 | 27.79 | −0.58 | 0.0184 |
| ENSG00000189184 | PCDH18 | 17.22 | 25.04 | 0.54 | 0.0184 |
| ENSG00000129667 | RHBDF2 | 1.64 | 3.22 | 0.97 | 0.0186 |
| ENSG00000152661 | GJA1 | 26.23 | 17.97 | −0.55 | 0.0189 |
| ENSG00000149968 | MMP3 | 217.23 | 120.92 | −0.85 | 0.019 |
| ENSG00000095303 | PTGS1 | 14.94 | 9.43 | −0.66 | 0.0191 |
| ENSG00000237094 | RP4−669L17.10 | 4.15 | 2.25 | −0.88 | 0.0192 |
| ENSG00000074410 | CA12 | 10.63 | 6.76 | −0.65 | 0.0202 |
| ENSG00000188185 | LINC00265 | 1.14 | 2.84 | 1.31 | 0.0205 |
| ENSG00000276368 | HIST1H2AJ | 113.82 | 75.57 | −0.59 | 0.0208 |
| ENSG00000255090 | MIR100HG | 3.93 | 1.35 | −1.55 | 0.0209 |
| ENSG00000081870 | HSPB11 | 58.78 | 40.08 | −0.55 | 0.021 |
| ENSG00000231890 | DARS−AS1 | 1.18 | 2.36 | 0.99 | 0.0213 |
| ENSG00000235173 | HGH1 | 1.53 | 3.09 | 1.02 | 0.0225 |
| ENSG00000156535 | CD109 | 13.57 | 9.51 | −0.51 | 0.0227 |
| ENSG00000101888 | NXT2 | 46.78 | 33.28 | −0.49 | 0.02325 |
| ENSG00000132010 | ZNF20 | 1.07 | 2.70 | 1.34 | 0.02345 |
| ENSG00000125968 | ID1 | 106.55 | 74.68 | −0.51 | 0.02355 |
| ENSG00000156113 | KCNMA1 | 36.54 | 25.24 | −0.53 | 0.0238 |
| ENSG00000269926 | RP11−442H21.2 | 4.00 | 8.01 | 1.00 | 0.0254 |
| ENSG00000135926 | TMBIM1 | 1.73 | 3.07 | 0.83 | 0.02565 |
| ENSG00000260518 | BMS1P8 | 2.50 | 5.10 | 1.03 | 0.02615 |
| ENSG00000170955 | PRKCDBP | 4.97 | 2.63 | −0.92 | 0.02635 |
| ENSG00000215126 | CBWD7 | 2.68 | 0.79 | −1.76 | 0.02695 |
| ENSG00000173801 | JUP | 1.05 | 1.96 | 0.90 | 0.0271 |
| ENSG00000173531 | MST1 | 0.94 | 1.94 | 1.06 | 0.02715 |
| ENSG00000136810 | TXN | 162.04 | 116.88 | −0.47 | 0.0273 |
| ENSG00000170961 | HAS2 | 29.97 | 20.55 | −0.54 | 0.02755 |
| ENSG00000116584 | ARHGEF2 | 18.10 | 30.25 | 0.74 | 0.02785 |
| ENSG00000164251 | F2RL1 | 14.68 | 9.94 | −0.56 | 0.03 |
| ENSG00000106330 | MOSPD3 | 3.07 | 1.54 | −0.99 | 0.0303 |
| ENSG00000164946 | FREM1 | 1.83 | 3.43 | 0.91 | 0.0309 |
| ENSG00000083845 | RPS5 | 130.60 | 186.23 | 0.51 | 0.0317 |
| ENSG00000123496 | IL13RA2 | 1.79 | 3.79 | 1.09 | 0.0319 |
| ENSG00000141956 | PRDM15 | 1.80 | 3.70 | 1.04 | 0.03215 |
| ENSG00000015133 | CCDC88C | 1.41 | 3.05 | 1.11 | 0.03215 |
| ENSG00000180573 | HIST1H2AC | 22.03 | 31.53 | 0.52 | 0.0329 |
| ENSG00000172020 | GAP43 | 100.45 | 73.22 | −0.46 | 0.03385 |
| ENSG00000048052 | HDAC9 | 38.82 | 56.35 | 0.54 | 0.0347 |
| ENSG00000262406 | MMP12 | 6.09 | 3.70 | −0.72 | 0.03485 |
| ENSG00000112902 | SEMA5A | 2.57 | 4.03 | 0.65 | 0.0349 |
| ENSG00000065833 | ME1 | 14.18 | 10.06 | −0.50 | 0.03505 |
| ENSG00000112769 | LAMA4 | 8.04 | 12.36 | 0.62 | 0.03505 |
| ENSG00000177606 | JUN | 8.69 | 12.64 | 0.54 | 0.03605 |
| ENSG00000179818 | PCBP1−AS1 | 12.27 | 17.00 | 0.47 | 0.03705 |
| ENSG00000187688 | TRPV2 | 0.87 | 1.90 | 1.13 | 0.03745 |
| ENSG00000144619 | CNTN4 | 2.80 | 5.02 | 0.85 | 0.0375 |
| ENSG00000165029 | ABCA1 | 3.37 | 4.75 | 0.50 | 0.03805 |
| ENSG00000133874 | RNF122 | 2.18 | 3.71 | 0.77 | 0.03825 |
| ENSG00000133884 | DPF2 | 13.03 | 20.47 | 0.65 | 0.0384 |
| ENSG00000204160 | ZDHHC18 | 3.15 | 6.17 | 0.97 | 0.03865 |
| ENSG00000276168 | RN7SL1 | 969.35 | 1711.54 | 0.82 | 0.03875 |
| ENSG00000010278 | CD9 | 39.01 | 28.37 | −0.46 | 0.03915 |
| ENSG00000198863 | RUNDC1 | 3.16 | 4.70 | 0.57 | 0.03935 |
| ENSG00000179115 | FARSA | 18.94 | 27.43 | 0.53 | 0.03965 |
| ENSG00000077713 | SLC25A43 | 7.44 | 4.98 | −0.58 | 0.03985 |
| ENSG00000184305 | CCSER1 | 3.29 | 5.16 | 0.65 | 0.03985 |
| ENSG00000118855 | MFSD1 | 34.06 | 24.12 | −0.50 | 0.04135 |
| ENSG00000186642 | PDE2A | 1.93 | 3.41 | 0.82 | 0.042 |
| ENSG00000021762 | OSBPL5 | 0.97 | 1.87 | 0.94 | 0.0421 |
| ENSG00000144655 | CSRNP1 | 1.66 | 2.83 | 0.77 | 0.04225 |
| ENSG00000167302 | ENTHD2 | 1.02 | 2.10 | 1.04 | 0.04295 |
| ENSG00000003147 | ICA1 | 52.16 | 78.32 | 0.59 | 0.0433 |
| ENSG00000104368 | PLAT | 38.86 | 61.97 | 0.67 | 0.0433 |
| ENSG00000197956 | S100A6 | 325.09 | 237.49 | −0.45 | 0.0435 |
| ENSG00000123609 | NMI | 3.10 | 1.75 | −0.83 | 0.04365 |
| ENSG00000175600 | SUGCT | 8.19 | 14.62 | 0.84 | 0.04365 |
| ENSG00000255121 | RP11−110I1.12 | 4.39 | 2.01 | −1.12 | 0.04425 |
| ENSG00000127586 | CHTF18 | 2.28 | 1.10 | −1.05 | 0.04435 |
| ENSG00000144674 | GOLGA4 | 45.93 | 31.67 | −0.54 | 0.0446 |
| ENSG00000214944 | ARHGEF28 | 3.83 | 6.08 | 0.67 | 0.0448 |
| ENSG00000182179 | UBA7 | 0.96 | 2.07 | 1.11 | 0.0449 |
| ENSG00000182752 | PAPPA | 30.32 | 19.39 | −0.65 | 0.04505 |
| ENSG00000006459 | KDM7A | 4.17 | 6.63 | 0.67 | 0.04515 |
| ENSG00000005238 | FAM214B | 3.78 | 5.72 | 0.60 | 0.0454 |
| ENSG00000011422 | PLAUR | 29.07 | 18.89 | −0.62 | 0.0459 |
| ENSG00000189410 | SH2D5 | 1.00 | 2.08 | 1.06 | 0.0461 |
| ENSG00000197614 | MFAP5 | 2.81 | 5.07 | 0.85 | 0.04635 |
| ENSG00000140464 | PML | 1.97 | 3.46 | 0.81 | 0.04685 |
| ENSG00000274070 | GATSL2 | 1.86 | 3.56 | 0.94 | 0.04715 |
| ENSG00000197345 | MRPL21 | 72.64 | 52.46 | −0.47 | 0.0473 |
| ENSG00000157379 | DHRS1 | 2.16 | 4.73 | 1.13 | 0.04735 |
| ENSG00000166341 | DCHS1 | 2.78 | 3.87 | 0.48 | 0.0479 |
| ENSG00000166133 | RPUSD2 | 3.01 | 4.76 | 0.66 | 0.0485 |
| ENSG00000148737 | TCF7L2 | 6.66 | 3.81 | −0.80 | 0.0495 |
| ENSG00000154556 | SORBS2 | 2.93 | 4.34 | 0.57 | 0.04995 |

| **SK-N-SH 72 h** | |  |  |  |  |
| --- | --- | --- | --- | --- | --- |
| **Gene ID** | **Gene** | **Value_1** | **Value_2** | **log2 (fold_change)** | ***p* value** |
| ENSG00000278771 | Metazoa_SRP | 124.23 | 28.35 | −2.13 | 5.00E−05 |
| ENSG00000277027 | RMRP | 1972.49 | 555.71 | −1.83 | 5.00E−05 |
| ENSG00000140859 | KIFC3 | 2.14 | 0.71 | −1.60 | 5.00E−05 |
| ENSG00000179950 | PUF60 | 8.45 | 3.41 | −1.31 | 5.00E−05 |
| ENSG00000173264 | GPR137 | 2.62 | 0.79 | −1.72 | 0.00015 |
| ENSG00000172663 | TMEM134 | 2.11 | 0.54 | −1.96 | 0.00025 |
| ENSG00000225663 | FAM195B | 2.86 | 0.78 | −1.87 | 0.00025 |
| ENSG00000167645 | YIF1B | 7.60 | 2.39 | −1.67 | 0.00025 |
| ENSG00000115268 | RPS15 | 59.59 | 23.04 | −1.37 | 0.00025 |
| ENSG00000008710 | PKD1 | 3.52 | 1.28 | −1.46 | 0.0003 |
| ENSG00000161016 | RPL8 | 232.03 | 109.85 | −1.08 | 0.0003 |
| ENSG00000169738 | DCXR | 9.41 | 2.82 | −1.74 | 0.00035 |
| ENSG00000130255 | RPL36 | 126.67 | 47.39 | −1.42 | 0.00035 |
| ENSG00000170889 | RPS9 | 64.10 | 25.89 | −1.31 | 0.0004 |
| ENSG00000173486 | FKBP2 | 15.90 | 5.58 | −1.51 | 0.0005 |
| ENSG00000277209 | RPPH1 | 912.26 | 392.97 | −1.22 | 0.0005 |
| ENSG00000169750 | RAC3 | 6.49 | 1.60 | −2.02 | 0.0006 |
| ENSG00000105499 | PLA2G4C | 4.37 | 1.54 | −1.51 | 0.0006 |
| ENSG00000101210 | EEF1A2 | 9.86 | 3.78 | −1.38 | 0.00065 |
| ENSG00000077080 | ACTL6B | 7.67 | 2.13 | −1.85 | 0.0007 |
| ENSG00000128564 | VGF | 18.20 | 6.99 | −1.38 | 0.00075 |
| ENSG00000050820 | BCAR1 | 2.32 | 0.66 | −1.82 | 0.0008 |
| ENSG00000274012 | Metazoa_SRP | 1774.01 | 854.12 | −1.05 | 0.001 |
| ENSG00000007384 | RHBDF1 | 2.98 | 0.86 | −1.79 | 0.0015 |
| ENSG00000162910 | MRPL55 | 5.16 | 2.55 | −1.02 | 0.0015 |
| ENSG00000123560 | PLP1 | 5.73 | 2.49 | −1.20 | 0.0016 |
| ENSG00000108691 | CCL2 | 114.22 | 56.58 | −1.01 | 0.00165 |
| ENSG00000168298 | HIST1H1E | 29.85 | 11.39 | −1.39 | 0.0017 |
| ENSG00000123358 | NR4A1 | 3.20 | 1.07 | −1.58 | 0.0018 |
| ENSG00000061938 | TNK2 | 2.37 | 0.77 | −1.62 | 0.0019 |
| ENSG00000274333 | RP11−717F1.2 | 0.80 | 2.40 | 1.59 | 0.0021 |
| ENSG00000197728 | RPS26 | 84.23 | 42.67 | −0.98 | 0.0022 |
| ENSG00000169696 | ASPSCR1 | 6.97 | 2.47 | −1.50 | 0.0023 |
| ENSG00000184260 | HIST2H2AC | 22.41 | 7.21 | −1.64 | 0.00235 |
| ENSG00000167468 | GPX4 | 36.56 | 16.98 | −1.11 | 0.0024 |
| ENSG00000184357 | HIST1H1B | 17.82 | 6.33 | −1.49 | 0.0025 |
| ENSG00000173457 | PPP1R14B | 47.32 | 22.38 | −1.08 | 0.0025 |
| ENSG00000127445 | PIN1 | 9.54 | 2.97 | −1.69 | 0.00255 |
| ENSG00000147403 | RPL10 | 326.55 | 172.52 | −0.92 | 0.00265 |
| ENSG00000160932 | LY6E | 5.27 | 1.99 | −1.40 | 0.00305 |
| ENSG00000084207 | GSTP1 | 50.71 | 27.07 | −0.91 | 0.0031 |
| ENSG00000100949 | RABGGTA | 2.27 | 0.88 | −1.36 | 0.00315 |
| ENSG00000100280 | AP1B1 | 6.88 | 3.37 | −1.03 | 0.00325 |
| ENSG00000276368 | HIST1H2AJ | 38.02 | 8.02 | −2.24 | 0.0033 |
| ENSG00000125534 | PPDPF | 4.24 | 1.11 | −1.93 | 0.0033 |
| ENSG00000134686 | PHC2 | 15.45 | 7.38 | −1.07 | 0.00345 |
| ENSG00000197879 | MYO1C | 17.63 | 8.46 | −1.06 | 0.0035 |
| ENSG00000214253 | FIS1 | 29.36 | 12.85 | −1.19 | 0.004 |
| ENSG00000145901 | TNIP1 | 12.71 | 4.96 | −1.36 | 0.00405 |
| ENSG00000104529 | EEF1D | 58.65 | 29.75 | −0.98 | 0.0042 |
| ENSG00000197457 | STMN3 | 8.96 | 4.50 | −0.99 | 0.00445 |
| ENSG00000175550 | DRAP1 | 28.17 | 13.42 | −1.07 | 0.00455 |
| ENSG00000186716 | BCR | 8.03 | 3.09 | −1.38 | 0.0046 |
| ENSG00000140988 | RPS2 | 598.78 | 334.28 | −0.84 | 0.0046 |
| ENSG00000103653 | CSK | 4.75 | 1.94 | −1.29 | 0.0047 |
| ENSG00000135390 | ATP5G2 | 70.96 | 38.64 | −0.88 | 0.0047 |
| ENSG00000120896 | SORBS3 | 3.13 | 1.23 | −1.35 | 0.0049 |
| ENSG00000075043 | KCNQ2 | 10.57 | 3.76 | −1.49 | 0.00495 |
| ENSG00000175592 | FOSL1 | 3.83 | 1.35 | −1.50 | 0.00515 |
| ENSG00000144589 | STK11IP | 3.18 | 1.15 | −1.46 | 0.00515 |
| ENSG00000268790 | CTC−429P9.4 | 6.04 | 0.54 | −3.48 | 0.0054 |
| ENSG00000171703 | TCEA2 | 6.90 | 2.56 | −1.43 | 0.00545 |
| ENSG00000165233 | C9orf89 | 2.73 | 0.82 | −1.74 | 0.0055 |
| ENSG00000126267 | COX6B1 | 137.41 | 74.44 | −0.88 | 0.0055 |
| ENSG00000154175 | ABI3BP | 2.36 | 1.10 | −1.10 | 0.0059 |
| ENSG00000106624 | AEBP1 | 12.77 | 5.21 | −1.29 | 0.00595 |
| ENSG00000161513 | FDXR | 2.95 | 1.13 | −1.39 | 0.006 |
| ENSG00000149091 | DGKZ | 3.30 | 1.26 | −1.38 | 0.006 |
| ENSG00000106683 | LIMK1 | 6.84 | 3.18 | −1.10 | 0.0062 |
| ENSG00000105552 | BCAT2 | 2.59 | 1.05 | −1.31 | 0.0063 |
| ENSG00000208037 | MIR320A | 197.05 | 22.91 | −3.10 | 0.0064 |
| ENSG00000011347 | SYT7 | 16.93 | 9.03 | −0.91 | 0.0064 |
| ENSG00000197971 | MBP | 4.27 | 2.40 | −0.83 | 0.0064 |
| ENSG00000110717 | NDUFS8 | 7.06 | 2.63 | −1.43 | 0.00665 |
| ENSG00000106628 | POLD2 | 21.18 | 11.43 | −0.89 | 0.0069 |
| ENSG00000204305 | AGER | 0.84 | 2.09 | 1.32 | 0.00715 |
| ENSG00000187840 | EIF4EBP1 | 11.91 | 4.77 | −1.32 | 0.00725 |
| ENSG00000013306 | SLC25A39 | 11.39 | 6.22 | −0.87 | 0.00765 |
| ENSG00000163931 | TKT | 28.92 | 16.58 | −0.80 | 0.0077 |
| ENSG00000172354 | GNB2 | 14.17 | 7.01 | −1.01 | 0.00775 |
| ENSG00000197903 | HIST1H2BK | 18.56 | 6.66 | −1.48 | 0.0078 |
| ENSG00000129925 | TMEM8A | 3.53 | 1.12 | −1.65 | 0.0081 |
| ENSG00000255571 | LINC00925 | 2.17 | 0.75 | −1.54 | 0.0081 |
| ENSG00000083444 | PLOD1 | 18.65 | 9.90 | −0.91 | 0.00835 |
| ENSG00000278588 | HIST1H2BI | 24.85 | 10.60 | −1.23 | 0.00855 |
| ENSG00000158863 | FAM160B2 | 3.91 | 1.32 | −1.56 | 0.0086 |
| ENSG00000133027 | PEMT | 3.61 | 1.41 | −1.35 | 0.00875 |
| ENSG00000127054 | CPSF3L | 16.48 | 7.40 | −1.16 | 0.009 |
| ENSG00000197122 | SRC | 7.43 | 3.51 | −1.08 | 0.0093 |
| ENSG00000183401 | CCDC159 | 3.86 | 1.31 | −1.56 | 0.00975 |
| ENSG00000126432 | PRDX5 | 61.22 | 32.69 | −0.90 | 0.01025 |
| ENSG00000105700 | KXD1 | 18.72 | 7.59 | −1.30 | 0.0105 |
| ENSG00000123143 | PKN1 | 9.20 | 4.53 | −1.02 | 0.01065 |
| ENSG00000061273 | HDAC7 | 6.07 | 3.01 | −1.01 | 0.01065 |
| ENSG00000117411 | B4GALT2 | 8.35 | 4.20 | −0.99 | 0.01065 |
| ENSG00000276966 | HIST1H4E | 150.64 | 84.00 | −0.84 | 0.01065 |
| ENSG00000136848 | DAB2IP | 2.84 | 1.38 | −1.04 | 0.01095 |
| ENSG00000163814 | CDCP1 | 5.48 | 2.62 | −1.07 | 0.01105 |
| ENSG00000072786 | STK10 | 6.01 | 2.59 | −1.22 | 0.0112 |
| ENSG00000108852 | MPP2 | 2.03 | 1.12 | −0.86 | 0.0114 |
| ENSG00000169727 | GPS1 | 14.24 | 8.51 | −0.74 | 0.01145 |
| ENSG00000177728 | KIAA0195 | 13.50 | 5.64 | −1.26 | 0.0115 |
| ENSG00000167526 | RPL13 | 213.29 | 126.64 | −0.75 | 0.0116 |
| ENSG00000196547 | MAN2A2 | 7.91 | 3.89 | −1.02 | 0.0118 |
| ENSG00000274267 | HIST1H3B | 46.48 | 22.88 | −1.02 | 0.0118 |
| ENSG00000083845 | RPS5 | 78.82 | 46.11 | −0.77 | 0.0118 |
| ENSG00000189057 | FAM111B | 4.19 | 8.13 | 0.96 | 0.0122 |
| ENSG00000159640 | ACE | 13.54 | 6.79 | −0.99 | 0.01225 |
| ENSG00000105419 | MEIS3 | 7.38 | 3.57 | −1.05 | 0.0123 |
| ENSG00000124155 | PIGT | 26.38 | 14.36 | −0.88 | 0.0123 |
| ENSG00000087086 | FTL | 239.95 | 144.51 | −0.73 | 0.0125 |
| ENSG00000136877 | FPGS | 7.37 | 2.86 | −1.36 | 0.01255 |
| ENSG00000172819 | RARG | 6.15 | 3.59 | −0.78 | 0.01255 |
| ENSG00000100116 | GCAT | 2.70 | 0.91 | −1.58 | 0.01265 |
| ENSG00000167797 | CDK2AP2 | 3.21 | 1.01 | −1.66 | 0.0129 |
| ENSG00000102878 | HSF4 | 4.06 | 1.60 | −1.35 | 0.01295 |
| ENSG00000137309 | HMGA1 | 27.67 | 15.68 | −0.82 | 0.01355 |
| ENSG00000105221 | AKT2 | 30.28 | 14.21 | −1.09 | 0.01395 |
| ENSG00000102977 | ACD | 4.12 | 1.61 | −1.36 | 0.01405 |
| ENSG00000214021 | TTLL3 | 6.07 | 2.94 | −1.04 | 0.01405 |
| ENSG00000185130 | HIST1H2BL | 10.57 | 4.57 | −1.21 | 0.0142 |
| ENSG00000144579 | CTDSP1 | 4.22 | 2.15 | −0.97 | 0.01485 |
| ENSG00000108518 | PFN1 | 175.51 | 108.19 | −0.70 | 0.01495 |
| ENSG00000225840 | AC010970.2 | 203.39 | 103.45 | −0.98 | 0.01505 |
| ENSG00000175220 | ARHGAP1 | 12.56 | 5.77 | −1.12 | 0.01525 |
| ENSG00000105372 | RPS19 | 232.40 | 133.62 | −0.80 | 0.0157 |
| ENSG00000273620 | AL591856.1 | 2.63 | 0.00 | #NAME? | 0.01605 |
| ENSG00000185215 | TNFAIP2 | 3.12 | 1.03 | −1.60 | 0.0161 |
| ENSG00000123136 | DDX39A | 8.41 | 4.26 | −0.98 | 0.0163 |
| ENSG00000123384 | LRP1 | 6.98 | 3.00 | −1.22 | 0.0164 |
| ENSG00000122490 | PQLC1 | 1.92 | 0.83 | −1.21 | 0.01665 |
| ENSG00000143774 | GUK1 | 6.57 | 3.86 | −0.77 | 0.0169 |
| ENSG00000185803 | SLC52A2 | 3.31 | 1.21 | −1.45 | 0.0173 |
| ENSG00000213672 | NCKIPSD | 2.02 | 0.75 | −1.43 | 0.01735 |
| ENSG00000164889 | SLC4A2 | 5.14 | 2.54 | −1.02 | 0.01745 |
| ENSG00000159713 | TPPP3 | 6.49 | 2.88 | −1.17 | 0.0177 |
| ENSG00000130305 | NSUN5 | 2.34 | 0.98 | −1.26 | 0.0178 |
| ENSG00000277075 | HIST1H2AE | 11.64 | 3.79 | −1.62 | 0.0179 |
| ENSG00000090447 | TFAP4 | 2.24 | 0.92 | −1.28 | 0.0182 |
| ENSG00000119408 | NEK6 | 6.22 | 3.45 | −0.85 | 0.0182 |
| ENSG00000274641 | HIST1H2BO | 58.13 | 30.89 | −0.91 | 0.0183 |
| ENSG00000166341 | DCHS1 | 2.16 | 1.20 | −0.84 | 0.0183 |
| ENSG00000177380 | PPFIA3 | 2.03 | 0.69 | −1.57 | 0.0184 |
| ENSG00000105520 | LPPR2 | 2.19 | 1.06 | −1.05 | 0.0184 |
| ENSG00000100379 | KCTD17 | 4.20 | 2.00 | −1.07 | 0.01845 |
| ENSG00000276168 | RN7SL1 | 716.39 | 349.03 | −1.04 | 0.0187 |
| ENSG00000142534 | RPS11 | 1573.32 | 942.50 | −0.74 | 0.01875 |
| ENSG00000142002 | DPP9 | 16.09 | 8.02 | −1.00 | 0.01895 |
| ENSG00000116809 | ZBTB17 | 2.54 | 1.17 | −1.12 | 0.01905 |
| ENSG00000139182 | CLSTN3 | 12.15 | 5.42 | −1.17 | 0.0192 |
| ENSG00000169429 | CXCL8 | 10.34 | 4.65 | −1.15 | 0.0194 |
| ENSG00000130175 | PRKCSH | 36.90 | 21.15 | −0.80 | 0.0195 |
| ENSG00000156453 | PCDH1 | 2.54 | 0.99 | −1.36 | 0.01955 |
| ENSG00000174886 | NDUFA11 | 3.65 | 1.12 | −1.70 | 0.0197 |
| ENSG00000197238 | HIST1H4J | 8.07 | 1.85 | −2.13 | 0.01985 |
| ENSG00000103932 | RPAP1 | 3.94 | 1.92 | −1.04 | 0.01985 |
| ENSG00000072195 | SPEG | 4.70 | 2.16 | −1.12 | 0.0199 |
| ENSG00000175756 | AURKAIP1 | 2.19 | 0.91 | −1.27 | 0.02015 |
| ENSG00000122515 | ZMIZ2 | 5.32 | 2.36 | −1.17 | 0.0203 |
| ENSG00000197858 | GPAA1 | 2.96 | 1.38 | −1.10 | 0.02045 |
| ENSG00000276256 | AC011043.1 | 4.15 | 7.66 | 0.89 | 0.02045 |
| ENSG00000099904 | ZDHHC8 | 2.06 | 0.49 | −2.08 | 0.02075 |
| ENSG00000167657 | DAPK3 | 1.95 | 0.55 | −1.81 | 0.0209 |
| ENSG00000142676 | RPL11 | 1221.02 | 767.86 | −0.67 | 0.02115 |
| ENSG00000105063 | PPP6R1 | 14.39 | 6.53 | −1.14 | 0.02125 |
| ENSG00000138834 | MAPK8IP3 | 4.32 | 1.74 | −1.31 | 0.0213 |
| ENSG00000089157 | RPLP0 | 701.78 | 421.81 | −0.73 | 0.0214 |
| ENSG00000123454 | DBH | 23.28 | 12.65 | −0.88 | 0.0216 |
| ENSG00000165915 | SLC39A13 | 2.01 | 0.86 | −1.22 | 0.0217 |
| ENSG00000136271 | DDX56 | 27.84 | 15.32 | −0.86 | 0.02175 |
| ENSG00000169220 | RGS14 | 2.15 | 0.82 | −1.40 | 0.02195 |
| ENSG00000160211 | G6PD | 8.12 | 3.79 | −1.10 | 0.02205 |
| ENSG00000174775 | HRAS | 3.01 | 1.26 | −1.25 | 0.0221 |
| ENSG00000105875 | WDR91 | 2.45 | 1.08 | −1.18 | 0.0222 |
| ENSG00000060656 | PTPRU | 4.18 | 2.04 | −1.03 | 0.02225 |
| ENSG00000103005 | USB1 | 5.35 | 2.81 | −0.93 | 0.02245 |
| ENSG00000162729 | IGSF8 | 2.33 | 1.15 | −1.02 | 0.02265 |
| ENSG00000063244 | U2AF2 | 14.69 | 8.99 | −0.71 | 0.0227 |
| ENSG00000102901 | CENPT | 5.20 | 2.43 | −1.10 | 0.0232 |
| ENSG00000174151 | CYB561D1 | 2.62 | 1.10 | −1.25 | 0.02325 |
| ENSG00000104915 | STX10 | 11.27 | 6.41 | −0.81 | 0.0234 |
| ENSG00000179094 | PER1 | 2.59 | 1.08 | −1.27 | 0.02345 |
| ENSG00000126458 | RRAS | 4.38 | 1.71 | −1.35 | 0.02355 |
| ENSG00000149923 | PPP4C | 10.50 | 5.78 | −0.86 | 0.02355 |
| ENSG00000200156 | RNU5B−1 | 12.58 | 30.92 | 1.30 | 0.02355 |
| ENSG00000163507 | KIAA1524 | 12.94 | 21.03 | 0.70 | 0.02365 |
| ENSG00000102103 | PQBP1 | 8.03 | 3.54 | −1.18 | 0.0237 |
| ENSG00000125991 | ERGIC3 | 101.58 | 60.16 | −0.76 | 0.0237 |
| ENSG00000149782 | PLCB3 | 4.46 | 2.52 | −0.82 | 0.0239 |
| ENSG00000230615 | RP5−1198O20.4 | 0.98 | 2.06 | 1.07 | 0.02395 |
| ENSG00000186010 | NDUFA13 | 13.62 | 4.80 | −1.51 | 0.02405 |
| ENSG00000130165 | ELOF1 | 19.15 | 10.31 | −0.89 | 0.02425 |
| ENSG00000160345 | C9orf116 | 254.95 | 147.51 | −0.79 | 0.0246 |
| ENSG00000100359 | SGSM3 | 2.79 | 0.90 | −1.62 | 0.02475 |
| ENSG00000185033 | SEMA4B | 3.61 | 1.71 | −1.08 | 0.0248 |
| ENSG00000160445 | ZER1 | 3.51 | 1.65 | −1.09 | 0.02495 |
| ENSG00000153406 | NMRAL1 | 10.94 | 4.12 | −1.41 | 0.02505 |
| ENSG00000105404 | RABAC1 | 6.30 | 3.06 | −1.04 | 0.02535 |
| ENSG00000105409 | ATP1A3 | 9.27 | 5.47 | −0.76 | 0.02555 |
| ENSG00000177697 | CD151 | 11.48 | 6.40 | −0.84 | 0.02565 |
| ENSG00000278962 | RP11−399B17.1 | 1.28 | 2.68 | 1.06 | 0.02575 |
| ENSG00000161999 | JMJD8 | 8.07 | 3.14 | −1.36 | 0.02585 |
| ENSG00000072071 | ADGRL1 | 2.85 | 1.15 | −1.31 | 0.02585 |
| ENSG00000107833 | NPM3 | 9.89 | 4.06 | −1.29 | 0.0259 |
| ENSG00000164587 | RPS14 | 297.37 | 186.94 | −0.67 | 0.0259 |
| ENSG00000187624 | C17orf97 | 2.91 | 1.28 | −1.18 | 0.02595 |
| ENSG00000205250 | E2F4 | 15.99 | 9.30 | −0.78 | 0.0261 |
| ENSG00000117713 | ARID1A | 17.28 | 9.97 | −0.79 | 0.0265 |
| ENSG00000118495 | PLAGL1 | 2.24 | 1.13 | −0.99 | 0.02665 |
| ENSG00000097021 | ACOT7 | 7.87 | 4.35 | −0.86 | 0.02665 |
| ENSG00000169100 | SLC25A6 | 44.29 | 28.25 | −0.65 | 0.0271 |
| ENSG00000130731 | C16orf13 | 7.39 | 3.85 | −0.94 | 0.02735 |
| ENSG00000108107 | RPL28 | 147.41 | 83.13 | −0.83 | 0.02735 |
| ENSG00000135441 | BLOC1S1 | 17.88 | 8.17 | −1.13 | 0.0276 |
| ENSG00000106333 | PCOLCE | 74.42 | 46.91 | −0.67 | 0.0278 |
| ENSG00000231890 | DARS−AS1 | 2.14 | 1.17 | −0.88 | 0.02785 |
| ENSG00000100906 | NFKBIA | 25.07 | 14.73 | −0.77 | 0.0282 |
| ENSG00000108604 | SMARCD2 | 32.51 | 21.09 | −0.62 | 0.02825 |
| ENSG00000167965 | MLST8 | 2.44 | 1.39 | −0.81 | 0.02845 |
| ENSG00000132879 | FBXO44 | 2.25 | 0.89 | −1.34 | 0.02855 |
| ENSG00000198837 | DENND4B | 4.81 | 2.62 | −0.87 | 0.02865 |
| ENSG00000186501 | TMEM222 | 2.85 | 1.26 | −1.18 | 0.02875 |
| ENSG00000274070 | GATSL2 | 2.10 | 0.87 | −1.27 | 0.0288 |
| ENSG00000197756 | RPL37A | 1343.44 | 816.13 | −0.72 | 0.0288 |
| ENSG00000262814 | MRPL12 | 8.63 | 4.12 | −1.07 | 0.02895 |
| ENSG00000108679 | LGALS3BP | 67.89 | 43.78 | −0.63 | 0.0297 |
| ENSG00000104884 | ERCC2 | 6.87 | 2.82 | −1.28 | 0.0298 |
| ENSG00000207205 | RNVU1−15 | 23.92 | 11.21 | −1.09 | 0.0299 |
| ENSG00000103148 | NPRL3 | 2.95 | 1.32 | −1.15 | 0.03025 |
| ENSG00000134594 | RAB33A | 3.74 | 1.78 | −1.07 | 0.03045 |
| ENSG00000111676 | ATN1 | 4.51 | 2.61 | −0.79 | 0.0309 |
| ENSG00000178896 | EXOSC4 | 2.92 | 0.96 | −1.60 | 0.031 |
| ENSG00000076604 | TRAF4 | 8.37 | 4.14 | −1.02 | 0.03155 |
| ENSG00000155363 | MOV10 | 8.03 | 3.93 | −1.03 | 0.0317 |
| ENSG00000110400 | PVRL1 | 3.97 | 1.91 | −1.06 | 0.03205 |
| ENSG00000100364 | KIAA0930 | 8.60 | 5.04 | −0.77 | 0.0322 |
| ENSG00000157350 | ST3GAL2 | 3.62 | 1.92 | −0.91 | 0.0323 |
| ENSG00000141540 | TTYH2 | 2.15 | 1.04 | −1.05 | 0.0324 |
| ENSG00000178982 | EIF3K | 40.83 | 25.29 | −0.69 | 0.0324 |
| ENSG00000141985 | SH3GL1 | 2.83 | 1.39 | −1.03 | 0.03255 |
| ENSG00000213976 | CTD−2561J22.2 | 0.91 | 2.07 | 1.19 | 0.03275 |
| ENSG00000027847 | B4GALT7 | 1.88 | 0.80 | −1.24 | 0.0329 |
| ENSG00000241360 | PDXP | 8.13 | 4.30 | −0.92 | 0.0329 |
| ENSG00000110057 | UNC93B1 | 1.86 | 0.74 | −1.33 | 0.03335 |
| ENSG00000126457 | PRMT1 | 43.57 | 26.76 | −0.70 | 0.03375 |
| ENSG00000106400 | ZNHIT1 | 37.79 | 21.44 | −0.82 | 0.03385 |
| ENSG00000102003 | SYP | 11.13 | 7.01 | −0.67 | 0.03395 |
| ENSG00000149476 | TKFC | 7.73 | 3.69 | −1.07 | 0.0342 |
| ENSG00000239779 | WBP1 | 27.30 | 15.53 | −0.81 | 0.03425 |
| ENSG00000164880 | INTS1 | 3.95 | 2.06 | −0.94 | 0.03445 |
| ENSG00000149295 | DRD2 | 1.97 | 0.82 | −1.27 | 0.03455 |
| ENSG00000100075 | SLC25A1 | 5.36 | 2.97 | −0.86 | 0.03455 |
| ENSG00000197905 | TEAD4 | 4.40 | 2.44 | −0.85 | 0.03455 |
| ENSG00000172725 | CORO1B | 2.50 | 1.13 | −1.15 | 0.03475 |
| ENSG00000106665 | CLIP2 | 4.46 | 2.45 | −0.86 | 0.03475 |
| ENSG00000198829 | SUCNR1 | 23.14 | 37.96 | 0.71 | 0.0349 |
| ENSG00000063180 | CA11 | 7.05 | 3.84 | −0.88 | 0.0351 |
| ENSG00000100304 | TTLL12 | 2.81 | 1.32 | −1.09 | 0.0353 |
| ENSG00000064692 | SNCAIP | 0.75 | 2.99 | 1.99 | 0.0353 |
| ENSG00000158158 | CNNM4 | 3.03 | 1.63 | −0.89 | 0.03575 |
| ENSG00000105568 | PPP2R1A | 34.07 | 19.90 | −0.78 | 0.03605 |
| ENSG00000122756 | CNTFR | 2.31 | 0.95 | −1.28 | 0.0361 |
| ENSG00000142186 | SCYL1 | 5.80 | 2.49 | −1.22 | 0.03615 |
| ENSG00000169223 | LMAN2 | 18.36 | 9.81 | −0.90 | 0.03655 |
| ENSG00000178188 | SH2B1 | 2.76 | 1.34 | −1.04 | 0.0366 |
| ENSG00000148303 | RPL7A | 818.49 | 530.73 | −0.62 | 0.03695 |
| ENSG00000248098 | BCKDHA | 4.57 | 1.41 | −1.69 | 0.0374 |
| ENSG00000108840 | HDAC5 | 3.33 | 1.35 | −1.31 | 0.0374 |
| ENSG00000239697 | TNFSF12 | 2.13 | 0.63 | −1.77 | 0.03755 |
| ENSG00000169992 | NLGN2 | 5.74 | 3.61 | −0.67 | 0.03755 |
| ENSG00000161558 | TMEM143 | 2.25 | 1.13 | −0.99 | 0.0378 |
| ENSG00000204536 | CCHCR1 | 3.96 | 2.24 | −0.82 | 0.03785 |
| ENSG00000200488 | RN7SKP203 | 5.27 | 1.23 | −2.09 | 0.03795 |
| ENSG00000160691 | SHC1 | 130.39 | 66.60 | −0.97 | 0.0381 |
| ENSG00000090339 | ICAM1 | 4.87 | 2.45 | −0.99 | 0.0382 |
| ENSG00000154134 | ROBO3 | 2.83 | 1.56 | −0.86 | 0.0386 |
| ENSG00000149806 | FAU | 156.24 | 85.42 | −0.87 | 0.0389 |
| ENSG00000025770 | NCAPH2 | 4.32 | 1.84 | −1.23 | 0.039 |
| ENSG00000141480 | ARRB2 | 7.98 | 5.01 | −0.67 | 0.039 |
| ENSG00000088882 | CPXM1 | 12.34 | 7.69 | −0.68 | 0.04055 |
| ENSG00000149260 | CAPN5 | 3.52 | 2.03 | −0.80 | 0.04075 |
| ENSG00000167654 | ATCAY | 9.85 | 6.08 | −0.70 | 0.0408 |
| ENSG00000167393 | PPP2R3B | 3.69 | 1.70 | −1.11 | 0.0409 |
| ENSG00000166165 | CKB | 22.00 | 12.56 | −0.81 | 0.041 |
| ENSG00000275713 | HIST1H2BH | 33.89 | 19.11 | −0.83 | 0.0411 |
| ENSG00000104853 | CLPTM1 | 14.90 | 8.29 | −0.85 | 0.04115 |
| ENSG00000105426 | PTPRS | 17.50 | 11.82 | −0.57 | 0.0412 |
| ENSG00000136824 | SMC2 | 19.92 | 30.56 | 0.62 | 0.04145 |
| ENSG00000080189 | SLC35C2 | 7.54 | 4.06 | −0.89 | 0.0415 |
| ENSG00000117682 | DHDDS | 11.12 | 7.07 | −0.65 | 0.0418 |
| ENSG00000146701 | MDH2 | 65.09 | 41.81 | −0.64 | 0.04185 |
| ENSG00000119688 | ABCD4 | 7.84 | 4.59 | −0.77 | 0.04195 |
| ENSG00000129667 | RHBDF2 | 1.87 | 0.96 | −0.97 | 0.04245 |
| ENSG00000163497 | FEV | 3.20 | 1.62 | −0.98 | 0.0426 |
| ENSG00000196155 | PLEKHG4 | 3.69 | 1.58 | −1.22 | 0.04275 |
| ENSG00000044090 | CUL7 | 5.02 | 2.71 | −0.89 | 0.04275 |
| ENSG00000103507 | BCKDK | 4.79 | 2.80 | −0.78 | 0.0428 |
| ENSG00000138160 | KIF11 | 17.32 | 26.68 | 0.62 | 0.04305 |
| ENSG00000105229 | PIAS4 | 3.18 | 1.43 | −1.15 | 0.0431 |
| ENSG00000163131 | CTSS | 5.12 | 1.99 | −1.36 | 0.04325 |
| ENSG00000142173 | COL6A2 | 6.57 | 2.75 | −1.26 | 0.04325 |
| ENSG00000032444 | PNPLA6 | 9.43 | 4.84 | −0.96 | 0.0437 |
| ENSG00000278099 | U1 | 0.14 | 1.92 | 3.80 | 0.0437 |
| ENSG00000198858 | R3HDM4 | 4.10 | 2.17 | −0.92 | 0.04375 |
| ENSG00000211584 | SLC48A1 | 2.35 | 1.12 | −1.07 | 0.04385 |
| ENSG00000105605 | CACNG7 | 2.31 | 0.93 | −1.32 | 0.04395 |
| ENSG00000162337 | LRP5 | 1.98 | 0.71 | −1.49 | 0.044 |
| ENSG00000100439 | ABHD4 | 3.51 | 1.55 | −1.18 | 0.04415 |
| ENSG00000090971 | NAT14 | 7.74 | 3.72 | −1.06 | 0.04435 |
| ENSG00000142669 | SH3BGRL3 | 53.81 | 33.42 | −0.69 | 0.04435 |
| ENSG00000109193 | SULT1E1 | 16.70 | 28.22 | 0.76 | 0.04435 |
| ENSG00000177156 | TALDO1 | 38.58 | 21.40 | −0.85 | 0.0444 |
| ENSG00000101220 | C20orf27 | 4.93 | 2.51 | −0.97 | 0.04455 |
| ENSG00000203879 | GDI1 | 32.27 | 22.03 | −0.55 | 0.0446 |
| ENSG00000197070 | ARRDC1 | 3.83 | 1.34 | −1.51 | 0.04495 |
| ENSG00000070404 | FSTL3 | 3.45 | 1.82 | −0.92 | 0.045 |
| ENSG00000181396 | OGFOD3 | 5.65 | 2.81 | −1.01 | 0.0452 |
| ENSG00000116685 | KIAA2013 | 6.46 | 3.93 | −0.72 | 0.0454 |
| ENSG00000131408 | NR1H2 | 9.60 | 4.74 | −1.02 | 0.04555 |
| ENSG00000224699 | LAMTOR5−AS1 | 2.61 | 1.72 | −0.60 | 0.04565 |
| ENSG00000166482 | MFAP4 | 44.51 | 22.24 | −1.00 | 0.04595 |
| ENSG00000167291 | TBC1D16 | 10.49 | 6.69 | −0.65 | 0.0461 |
| ENSG00000107874 | CUEDC2 | 10.01 | 5.65 | −0.82 | 0.04635 |
| ENSG00000039650 | PNKP | 4.53 | 2.22 | −1.03 | 0.0464 |
| ENSG00000111678 | C12orf57 | 7.29 | 2.82 | −1.37 | 0.04655 |
| ENSG00000198400 | NTRK1 | 2.24 | 0.77 | −1.55 | 0.04675 |
| ENSG00000104960 | PTOV1 | 9.78 | 4.74 | −1.04 | 0.04675 |
| ENSG00000106077 | ABHD11 | 3.75 | 2.24 | −0.74 | 0.0473 |
| ENSG00000085117 | CD82 | 3.99 | 1.92 | −1.05 | 0.04765 |
| ENSG00000121653 | MAPK8IP1 | 5.22 | 3.15 | −0.73 | 0.04775 |
| ENSG00000103145 | HCFC1R1 | 14.21 | 7.17 | −0.99 | 0.04785 |
| ENSG00000072062 | PRKACA | 12.31 | 6.81 | −0.85 | 0.04785 |
| ENSG00000160213 | CSTB | 10.24 | 6.46 | −0.67 | 0.04785 |
| ENSG00000135926 | TMBIM1 | 2.90 | 1.62 | −0.84 | 0.0481 |
| ENSG00000248927 | CTD−2334D19.1 | 2.47 | 4.93 | 0.99 | 0.0484 |
| ENSG00000112312 | GMNN | 32.29 | 51.32 | 0.67 | 0.0485 |
| ENSG00000184828 | ZBTB7C | 2.47 | 1.51 | −0.71 | 0.04855 |
| ENSG00000213676 | ATF6B | 12.32 | 5.88 | −1.07 | 0.0487 |
| ENSG00000118162 | KPTN | 4.21 | 1.82 | −1.21 | 0.04875 |
| ENSG00000114115 | RBP1 | 83.09 | 51.83 | −0.68 | 0.04875 |
| ENSG00000130724 | CHMP2A | 58.61 | 38.06 | −0.62 | 0.0488 |
| ENSG00000130313 | PGLS | 3.94 | 1.42 | −1.47 | 0.04885 |
| ENSG00000167674 | HDGFRP2 | 8.61 | 4.35 | −0.98 | 0.0489 |
| ENSG00000089327 | FXYD5 | 12.44 | 6.90 | −0.85 | 0.04895 |
| ENSG00000162191 | UBXN1 | 64.08 | 39.03 | −0.72 | 0.04935 |
| ENSG00000105254 | TBCB | 68.57 | 42.66 | −0.68 | 0.0494 |
| ENSG00000107331 | ABCA2 | 2.31 | 1.14 | −1.01 | 0.04945 |
| ENSG00000005075 | POLR2J | 6.26 | 2.71 | −1.21 | 0.0496 |
| ENSG00000177963 | RIC8A | 17.13 | 11.00 | −0.64 | 0.0496 |
| ENSG00000124635 | HIST1H2BJ | 31.14 | 17.06 | −0.87 | 0.04965 |

| **ReNcell CX 24 h** | | |  | |  | |  |  |
| --- | --- | --- | --- | --- | --- | --- | --- | --- |
| **Gene ID** | **Gene** | **Value_1** | | **Value_2** | | **log2 (Fold change)** | | ***p* value** |
| ENSG00000227195 | MIR663AHG | 3.98 | | 10.84 | | 1.44 | | 5.00E−05 |
| ENSG00000118523 | CTGF | 66.05 | | 104.91 | | 0.67 | | 0.00015 |
| ENSG00000131126 | TEX101 | 1.92 | | 5.01 | | 1.39 | | 0.0002 |
| ENSG00000077463 | SIRT6 | 0.38 | | 1.24 | | 1.70 | | 0.00025 |
| ENSG00000126709 | IFI6 | 88.37 | | 56.42 | | −0.65 | | 0.00035 |
| ENSG00000157601 | MX1 | 39.17 | | 21.60 | | −0.86 | | 0.0005 |
| ENSG00000151012 | SLC7A11 | 8.14 | | 12.46 | | 0.61 | | 0.00055 |
| ENSG00000175592 | FOSL1 | 5.93 | | 11.76 | | 0.99 | | 0.00065 |
| ENSG00000263934 | SNORD3A | 1382.17 | | 951.95 | | −0.54 | | 0.0008 |
| ENSG00000130558 | OLFM1 | 0.54 | | 1.84 | | 1.78 | | 0.0008 |
| ENSG00000148053 | NTRK2 | 0.88 | | 1.69 | | 0.94 | | 0.0009 |
| ENSG00000279093 | RP11−856B14.10 | 1.50 | | 0.00 | | #NAME? | | 0.00115 |
| ENSG00000186642 | PDE2A | 0.69 | | 1.67 | | 1.28 | | 0.00125 |
| ENSG00000101096 | NFATC2 | 1.94 | | 3.27 | | 0.75 | | 0.0018 |
| ENSG00000120738 | EGR1 | 9.63 | | 14.68 | | 0.61 | | 0.00215 |
| ENSG00000070444 | MNT | 1.07 | | 2.22 | | 1.04 | | 0.0022 |
| ENSG00000131981 | LGALS3 | 253.36 | | 179.57 | | −0.50 | | 0.00235 |
| ENSG00000139645 | ANKRD52 | 6.41 | | 9.92 | | 0.63 | | 0.00235 |
| ENSG00000100393 | EP300 | 6.28 | | 9.05 | | 0.53 | | 0.00265 |
| ENSG00000163347 | CLDN1 | 2.11 | | 3.63 | | 0.78 | | 0.0029 |
| ENSG00000160345 | C9orf116 | 49.07 | | 80.74 | | 0.72 | | 0.00365 |
| ENSG00000162614 | NEXN | 4.05 | | 7.23 | | 0.84 | | 0.00375 |
| ENSG00000225840 | AC010970.2 | 32.67 | | 54.02 | | 0.73 | | 0.0039 |
| ENSG00000127481 | UBR4 | 15.62 | | 23.71 | | 0.60 | | 0.00405 |
| ENSG00000153815 | CMIP | 6.12 | | 9.30 | | 0.60 | | 0.0044 |
| ENSG00000113739 | STC2 | 3.62 | | 5.46 | | 0.59 | | 0.00445 |
| ENSG00000142871 | CYR61 | 23.31 | | 33.48 | | 0.52 | | 0.00475 |
| ENSG00000137965 | IFI44 | 17.13 | | 9.57 | | −0.84 | | 0.0048 |
| ENSG00000181827 | RFX7 | 8.64 | | 12.96 | | 0.59 | | 0.0049 |
| ENSG00000136997 | MYC | 84.93 | | 116.58 | | 0.46 | | 0.00495 |
| ENSG00000136295 | TTYH3 | 5.33 | | 7.90 | | 0.57 | | 0.00505 |
| ENSG00000121316 | PLBD1 | 1.63 | | 0.54 | | −1.61 | | 0.0051 |
| ENSG00000275216 | RP11−54H7.4 | 2.08 | | 3.51 | | 0.76 | | 0.00515 |
| ENSG00000274012 | Metazoa_SRP | 2989.47 | | 2150.18 | | −0.48 | | 0.0052 |
| ENSG00000128564 | VGF | 0.78 | | 1.83 | | 1.23 | | 0.0053 |
| ENSG00000103257 | SLC7A5 | 3.31 | | 5.16 | | 0.64 | | 0.00565 |
| ENSG00000153071 | DAB2 | 24.17 | | 35.15 | | 0.54 | | 0.0058 |
| ENSG00000198755 | RPL10A | 898.68 | | 662.39 | | −0.44 | | 0.0059 |
| ENSG00000102554 | KLF5 | 9.13 | | 15.84 | | 0.79 | | 0.0061 |
| ENSG00000111676 | ATN1 | 4.77 | | 7.33 | | 0.62 | | 0.00655 |
| ENSG00000120549 | KIAA1217 | 14.22 | | 20.79 | | 0.55 | | 0.0066 |
| ENSG00000204428 | LY6G5C | 0.49 | | 1.58 | | 1.68 | | 0.00675 |
| ENSG00000251141 | RP11−53O19.1 | 6.00 | | 2.47 | | −1.28 | | 0.0071 |
| ENSG00000064393 | HIPK2 | 11.42 | | 16.49 | | 0.53 | | 0.0071 |
| ENSG00000274281 | RP11−326N17.2 | 0.93 | | 2.02 | | 1.11 | | 0.00715 |
| ENSG00000125462 | C1orf61 | 0.68 | | 1.35 | | 0.98 | | 0.0072 |
| ENSG00000215012 | C22orf29 | 1.82 | | 2.92 | | 0.69 | | 0.0073 |
| ENSG00000185864 | NPIPB4 | 4.96 | | 10.35 | | 1.06 | | 0.00755 |
| ENSG00000224023 | FLJ37035 | 1.24 | | 0.17 | | −2.91 | | 0.0078 |
| ENSG00000157933 | SKI | 3.04 | | 5.27 | | 0.79 | | 0.0086 |
| ENSG00000122678 | POLM | 1.37 | | 2.66 | | 0.95 | | 0.00865 |
| ENSG00000122026 | RPL21 | 1212.77 | | 887.06 | | −0.45 | | 0.0087 |
| ENSG00000181222 | POLR2A | 12.09 | | 18.32 | | 0.60 | | 0.0089 |
| ENSG00000173660 | UQCRH | 303.21 | | 227.01 | | −0.42 | | 0.00905 |
| ENSG00000114019 | AMOTL2 | 11.08 | | 15.37 | | 0.47 | | 0.00905 |
| ENSG00000275342 | SGK223 | 1.19 | | 1.99 | | 0.74 | | 0.00905 |
| ENSG00000143614 | GATAD2B | 4.97 | | 6.99 | | 0.49 | | 0.0096 |
| ENSG00000110104 | CCDC86 | 5.84 | | 9.60 | | 0.72 | | 0.0096 |
| ENSG00000144711 | IQSEC1 | 1.64 | | 2.72 | | 0.73 | | 0.0098 |
| ENSG00000166896 | XRCC6BP1 | 4.59 | | 2.47 | | −0.89 | | 0.0099 |
| ENSG00000186468 | RPS23 | 1531.20 | | 1100.56 | | −0.48 | | 0.01005 |
| ENSG00000197956 | S100A6 | 703.49 | | 525.51 | | −0.42 | | 0.01005 |
| ENSG00000136715 | SAP130 | 5.05 | | 7.32 | | 0.54 | | 0.01005 |
| ENSG00000125356 | NDUFA1 | 57.71 | | 39.16 | | −0.56 | | 0.0101 |
| ENSG00000125945 | ZNF436 | 181.93 | | 135.21 | | −0.43 | | 0.01015 |
| ENSG00000166963 | MAP1A | 4.80 | | 6.65 | | 0.47 | | 0.01055 |
| ENSG00000137959 | IFI44L | 19.84 | | 11.57 | | −0.78 | | 0.0108 |
| ENSG00000143126 | CELSR2 | 1.89 | | 2.93 | | 0.64 | | 0.0108 |
| ENSG00000185885 | IFITM1 | 22.39 | | 13.75 | | −0.70 | | 0.012 |
| ENSG00000132639 | SNAP25 | 0.57 | | 3.37 | | 2.58 | | 0.0122 |
| ENSG00000200972 | RNU5A−8P | 55.24 | | 21.14 | | −1.39 | | 0.01305 |
| ENSG00000105894 | PTN | 211.35 | | 160.42 | | −0.40 | | 0.01335 |
| ENSG00000130382 | MLLT1 | 4.70 | | 6.69 | | 0.51 | | 0.0135 |
| ENSG00000276368 | HIST1H2AJ | 18.01 | | 29.29 | | 0.70 | | 0.0136 |
| ENSG00000129824 | RPS4Y1 | 355.95 | | 269.69 | | −0.40 | | 0.0138 |
| ENSG00000109475 | RPL34 | 1241.62 | | 938.78 | | −0.40 | | 0.01435 |
| ENSG00000221983 | UBA52 | 446.37 | | 335.87 | | −0.41 | | 0.0147 |
| ENSG00000080802 | CNOT4 | 7.26 | | 11.54 | | 0.67 | | 0.01495 |
| ENSG00000103460 | TOX3 | 6.08 | | 8.63 | | 0.50 | | 0.01545 |
| ENSG00000128342 | LIF | 2.22 | | 4.71 | | 1.08 | | 0.01625 |
| ENSG00000072135 | PTPN18 | 3.96 | | 6.13 | | 0.63 | | 0.0164 |
| ENSG00000163584 | RPL22L1 | 244.78 | | 182.38 | | −0.42 | | 0.0166 |
| ENSG00000053254 | FOXN3 | 11.56 | | 16.54 | | 0.52 | | 0.01705 |
| ENSG00000231500 | RPS18 | 1209.12 | | 910.01 | | −0.41 | | 0.0171 |
| ENSG00000173947 | PIFO | 1.63 | | 0.59 | | −1.48 | | 0.0173 |
| ENSG00000111653 | ING4 | 40.87 | | 27.69 | | −0.56 | | 0.0175 |
| ENSG00000100162 | CENPM | 1.41 | | 2.80 | | 1.00 | | 0.01755 |
| ENSG00000125398 | SOX9 | 27.36 | | 36.10 | | 0.40 | | 0.01795 |
| ENSG00000174943 | KCTD13 | 1.17 | | 2.60 | | 1.15 | | 0.01795 |
| ENSG00000172534 | HCFC1 | 3.83 | | 6.29 | | 0.72 | | 0.01835 |
| ENSG00000180370 | PAK2 | 21.92 | | 28.72 | | 0.39 | | 0.01865 |
| ENSG00000225914 | XXbac−BPG154L12.4 | 0.98 | | 1.66 | | 0.77 | | 0.01885 |
| ENSG00000171451 | DSEL | 1.92 | | 2.96 | | 0.62 | | 0.01935 |
| ENSG00000117226 | GBP3 | 17.73 | | 11.06 | | −0.68 | | 0.01975 |
| ENSG00000132361 | CLUH | 10.74 | | 21.61 | | 1.01 | | 0.0203 |
| ENSG00000162231 | NXF1 | 14.02 | | 22.61 | | 0.69 | | 0.0204 |
| ENSG00000163682 | RPL9 | 1489.04 | | 1113.07 | | −0.42 | | 0.02055 |
| ENSG00000182158 | CREB3L2 | 8.00 | | 10.76 | | 0.43 | | 0.0213 |
| ENSG00000230606 | AC159540.1 | 15.58 | | 22.84 | | 0.55 | | 0.0213 |
| ENSG00000110711 | AIP | 8.06 | | 4.92 | | −0.71 | | 0.0214 |
| ENSG00000168028 | RPSA | 565.93 | | 432.42 | | −0.39 | | 0.0214 |
| ENSG00000180071 | ANKRD18A | 1.20 | | 3.48 | | 1.53 | | 0.02145 |
| ENSG00000280165 | PCDH20 | 19.49 | | 14.85 | | −0.39 | | 0.02165 |
| ENSG00000179833 | SERTAD2 | 3.98 | | 5.60 | | 0.49 | | 0.0218 |
| ENSG00000224861 | YBX1P1 | 1.13 | | 0.42 | | −1.43 | | 0.0222 |
| ENSG00000134597 | RBMX2 | 31.95 | | 23.05 | | −0.47 | | 0.0222 |
| ENSG00000147604 | RPL7 | 1747.38 | | 1283.73 | | −0.44 | | 0.0222 |
| ENSG00000120942 | UBIAD1 | 2.82 | | 4.44 | | 0.66 | | 0.0222 |
| ENSG00000165271 | NOL6 | 18.09 | | 24.24 | | 0.42 | | 0.0225 |
| ENSG00000176171 | BNIP3 | 36.89 | | 26.37 | | −0.48 | | 0.02265 |
| ENSG00000172809 | RPL38 | 1058.90 | | 823.16 | | −0.36 | | 0.02315 |
| ENSG00000076716 | GPC4 | 13.86 | | 18.41 | | 0.41 | | 0.0233 |
| ENSG00000255823 | MTRNR2L8 | 6.46 | | 10.43 | | 0.69 | | 0.0233 |
| ENSG00000174059 | CD34 | 1.11 | | 2.03 | | 0.87 | | 0.02335 |
| ENSG00000172936 | MYD88 | 4.08 | | 5.88 | | 0.53 | | 0.02385 |
| ENSG00000260804 | PKI55 | 5.56 | | 7.82 | | 0.49 | | 0.02395 |
| ENSG00000008988 | RPS20 | 1563.47 | | 1193.12 | | −0.39 | | 0.024 |
| ENSG00000076944 | STXBP2 | 1.42 | | 0.59 | | −1.26 | | 0.02415 |
| ENSG00000277209 | RPPH1 | 2305.23 | | 1689.14 | | −0.45 | | 0.0243 |
| ENSG00000144218 | AFF3 | 0.63 | | 1.26 | | 1.00 | | 0.02515 |
| ENSG00000177954 | RPS27 | 1855.23 | | 1443.30 | | −0.36 | | 0.0256 |
| ENSG00000176978 | DPP7 | 7.67 | | 12.98 | | 0.76 | | 0.0258 |
| ENSG00000173212 | MAB21L3 | 2.06 | | 3.11 | | 0.59 | | 0.0259 |
| ENSG00000101849 | TBL1X | 4.38 | | 6.02 | | 0.46 | | 0.0261 |
| ENSG00000146592 | CREB5 | 7.61 | | 11.66 | | 0.62 | | 0.02625 |
| ENSG00000265681 | RPL17 | 2218.36 | | 1665.57 | | −0.41 | | 0.0263 |
| ENSG00000138326 | RPS24 | 3549.14 | | 2669.31 | | −0.41 | | 0.0265 |
| ENSG00000143333 | RGS16 | 8.77 | | 6.26 | | −0.49 | | 0.0267 |
| ENSG00000125730 | C3 | 3.00 | | 1.27 | | −1.24 | | 0.02675 |
| ENSG00000151065 | DCP1B | 5.73 | | 9.14 | | 0.67 | | 0.027 |
| ENSG00000165023 | DIRAS2 | 1.04 | | 1.68 | | 0.69 | | 0.02705 |
| ENSG00000119596 | YLPM1 | 14.79 | | 20.04 | | 0.44 | | 0.02715 |
| ENSG00000188021 | UBQLN2 | 6.02 | | 8.21 | | 0.45 | | 0.02715 |
| ENSG00000173402 | DAG1 | 17.24 | | 22.02 | | 0.35 | | 0.0273 |
| ENSG00000119632 | IFI27L2 | 17.81 | | 11.70 | | −0.61 | | 0.0274 |
| ENSG00000187994 | RINL | 3.26 | | 1.86 | | −0.81 | | 0.0276 |
| ENSG00000171863 | RPS7 | 871.49 | | 675.98 | | −0.37 | | 0.0286 |
| ENSG00000197958 | RPL12 | 1518.56 | | 1176.67 | | −0.37 | | 0.02875 |
| ENSG00000105880 | DLX5 | 5.29 | | 7.71 | | 0.54 | | 0.02895 |
| ENSG00000110237 | ARHGEF17 | 1.93 | | 3.04 | | 0.66 | | 0.0293 |
| ENSG00000148773 | MKI67 | 14.37 | | 19.03 | | 0.40 | | 0.02955 |
| ENSG00000229117 | RPL41 | 1200.43 | | 939.05 | | −0.35 | | 0.0299 |
| ENSG00000207205 | RNVU1−15 | 20.72 | | 11.55 | | −0.84 | | 0.0301 |
| ENSG00000155090 | KLF10 | 27.89 | | 35.89 | | 0.36 | | 0.0301 |
| ENSG00000267051 | RP11−128P17.2 | 1.24 | | 0.47 | | −1.42 | | 0.03015 |
| ENSG00000182378 | PLCXD1 | 0.94 | | 1.82 | | 0.95 | | 0.0303 |
| ENSG00000146707 | POMZP3 | 2.92 | | 5.93 | | 1.02 | | 0.0304 |
| ENSG00000169710 | FASN | 7.01 | | 9.39 | | 0.42 | | 0.03045 |
| ENSG00000151500 | THYN1 | 59.62 | | 45.01 | | −0.41 | | 0.03055 |
| ENSG00000127527 | EPS15L1 | 7.97 | | 11.18 | | 0.49 | | 0.03075 |
| ENSG00000005339 | CREBBP | 8.60 | | 15.51 | | 0.85 | | 0.03105 |
| ENSG00000139239 | RPL14P1 | 0.31 | | 1.20 | | 1.97 | | 0.03115 |
| ENSG00000122547 | EEPD1 | 3.76 | | 1.74 | | −1.11 | | 0.0313 |
| ENSG00000232164 | AC092669.3 | 3.99 | | 5.91 | | 0.57 | | 0.0314 |
| ENSG00000136160 | EDNRB | 19.31 | | 13.80 | | −0.49 | | 0.03145 |
| ENSG00000116251 | RPL22 | 503.50 | | 355.15 | | −0.50 | | 0.03155 |
| ENSG00000075702 | WDR62 | 0.66 | | 1.23 | | 0.90 | | 0.03155 |
| ENSG00000237037 | NDUFA6−AS1 | 1.38 | | 0.52 | | −1.40 | | 0.03165 |
| ENSG00000166716 | ZNF592 | 2.06 | | 3.27 | | 0.67 | | 0.03205 |
| ENSG00000196367 | TRRAP | 4.61 | | 6.38 | | 0.47 | | 0.0321 |
| ENSG00000145425 | RPS3A | 1625.89 | | 1216.98 | | −0.42 | | 0.0324 |
| ENSG00000149273 | RPS3 | 1466.25 | | 1130.94 | | −0.37 | | 0.03245 |
| ENSG00000087258 | GNAO1 | 0.88 | | 1.97 | | 1.16 | | 0.03265 |
| ENSG00000157657 | ZNF618 | 2.06 | | 2.89 | | 0.49 | | 0.03285 |
| ENSG00000108984 | MAP2K6 | 21.58 | | 15.37 | | −0.49 | | 0.0329 |
| ENSG00000126267 | COX6B1 | 153.09 | | 116.34 | | −0.40 | | 0.033 |
| ENSG00000167978 | SRRM2 | 77.84 | | 131.35 | | 0.75 | | 0.0334 |
| ENSG00000034510 | TMSB10 | 1605.61 | | 1259.19 | | −0.35 | | 0.03365 |
| ENSG00000125538 | IL1B | 1.17 | | 0.51 | | −1.19 | | 0.0337 |
| ENSG00000148303 | RPL7A | 1359.66 | | 1049.01 | | −0.37 | | 0.03385 |
| ENSG00000249731 | RP11−259O2.3 | 10.04 | | 15.17 | | 0.60 | | 0.03385 |
| ENSG00000130479 | MAP1S | 1.87 | | 3.23 | | 0.79 | | 0.03385 |
| ENSG00000116017 | ARID3A | 1.35 | | 0.56 | | −1.26 | | 0.034 |
| ENSG00000277791 | PSMB3 | 86.54 | | 64.71 | | −0.42 | | 0.03405 |
| ENSG00000117448 | AKR1A1 | 68.31 | | 48.37 | | −0.50 | | 0.0342 |
| ENSG00000165795 | NDRG2 | 2.14 | | 3.74 | | 0.81 | | 0.03455 |
| ENSG00000108821 | COL1A1 | 7.47 | | 11.31 | | 0.60 | | 0.03465 |
| ENSG00000167470 | MIDN | 3.11 | | 4.38 | | 0.49 | | 0.0347 |
| ENSG00000120833 | SOCS2 | 15.03 | | 9.72 | | −0.63 | | 0.03475 |
| ENSG00000185585 | OLFML2A | 5.74 | | 7.64 | | 0.41 | | 0.035 |
| ENSG00000196531 | NACA | 820.48 | | 638.38 | | −0.36 | | 0.03515 |
| ENSG00000235978 | AC018816.3 | 1.35 | | 0.38 | | −1.84 | | 0.0353 |
| ENSG00000237857 | RP11−435O5.2 | 0.56 | | 1.17 | | 1.07 | | 0.03565 |
| ENSG00000256073 | URB1−AS1 | 6.78 | | 4.29 | | −0.66 | | 0.0357 |
| ENSG00000140948 | ZCCHC14 | 3.35 | | 4.58 | | 0.45 | | 0.0358 |
| ENSG00000137752 | CASP1 | 14.73 | | 9.29 | | −0.67 | | 0.0361 |
| ENSG00000126016 | AMOT | 7.09 | | 10.17 | | 0.52 | | 0.0364 |
| ENSG00000117450 | PRDX1 | 644.75 | | 502.13 | | −0.36 | | 0.03655 |
| ENSG00000165632 | TAF3 | 2.14 | | 3.04 | | 0.51 | | 0.03655 |
| ENSG00000149639 | SOGA1 | 2.28 | | 3.19 | | 0.48 | | 0.0369 |
| ENSG00000259380 | RP11−346D14.1 | 4.49 | | 6.38 | | 0.51 | | 0.037 |
| ENSG00000164442 | CITED2 | 19.75 | | 26.07 | | 0.40 | | 0.0372 |
| ENSG00000253103 | LINC01609 | 0.43 | | 1.23 | | 1.51 | | 0.0374 |
| ENSG00000089289 | IGBP1 | 32.39 | | 24.88 | | −0.38 | | 0.03745 |
| ENSG00000167925 | GHDC | 2.51 | | 1.56 | | −0.68 | | 0.0375 |
| ENSG00000188064 | WNT7B | 0.76 | | 1.29 | | 0.76 | | 0.03755 |
| ENSG00000100902 | PSMA6 | 276.31 | | 188.05 | | −0.56 | | 0.0378 |
| ENSG00000164236 | ANKRD33B | 1.31 | | 1.83 | | 0.49 | | 0.0379 |
| ENSG00000276168 | RN7SL1 | 1132.58 | | 801.71 | | −0.50 | | 0.03795 |
| ENSG00000123131 | PRDX4 | 109.66 | | 84.05 | | −0.38 | | 0.03815 |
| ENSG00000165912 | PACSIN3 | 1.95 | | 3.36 | | 0.79 | | 0.03825 |
| ENSG00000261325 | AC140542.2 | 1.47 | | 0.72 | | −1.02 | | 0.03855 |
| ENSG00000137806 | NDUFAF1 | 22.08 | | 15.92 | | −0.47 | | 0.03875 |
| ENSG00000072506 | HSD17B10 | 38.55 | | 27.04 | | −0.51 | | 0.0388 |
| ENSG00000104894 | CD37 | 0.49 | | 1.10 | | 1.17 | | 0.0389 |
| ENSG00000115271 | GCA | 3.39 | | 1.97 | | −0.78 | | 0.03925 |
| ENSG00000182899 | RPL35A | 966.55 | | 760.41 | | −0.35 | | 0.03925 |
| ENSG00000220848 | RPS18P9 | 2.06 | | 0.81 | | −1.34 | | 0.0394 |
| ENSG00000140092 | FBLN5 | 2.46 | | 4.33 | | 0.82 | | 0.03945 |
| ENSG00000233639 | LINC01158 | 20.19 | | 14.04 | | −0.52 | | 0.03955 |
| ENSG00000185650 | ZFP36L1 | 14.76 | | 19.20 | | 0.38 | | 0.0397 |
| ENSG00000184545 | DUSP8 | 0.34 | | 1.24 | | 1.87 | | 0.03975 |
| ENSG00000131711 | MAP1B | 115.89 | | 153.78 | | 0.41 | | 0.03985 |
| ENSG00000110395 | CBL | 7.87 | | 9.96 | | 0.34 | | 0.0401 |
| ENSG00000137507 | LRRC32 | 1.18 | | 0.44 | | −1.43 | | 0.0408 |
| ENSG00000166352 | C11orf74 | 15.75 | | 10.74 | | −0.55 | | 0.0409 |
| ENSG00000015475 | BID | 24.70 | | 18.73 | | −0.40 | | 0.0413 |
| ENSG00000065923 | SLC9A7 | 1.42 | | 2.07 | | 0.54 | | 0.04135 |
| ENSG00000229833 | PET100 | 48.17 | | 34.65 | | −0.48 | | 0.0414 |
| ENSG00000160741 | CRTC2 | 2.51 | | 5.31 | | 1.08 | | 0.04155 |
| ENSG00000107862 | GBF1 | 6.66 | | 8.70 | | 0.38 | | 0.04165 |
| ENSG00000170889 | RPS9 | 185.33 | | 142.45 | | −0.38 | | 0.04175 |
| ENSG00000167767 | KRT80 | 1.80 | | 2.63 | | 0.55 | | 0.04185 |
| ENSG00000115844 | DLX2 | 2.05 | | 3.09 | | 0.59 | | 0.04195 |
| ENSG00000187801 | ZFP69B | 3.19 | | 4.69 | | 0.55 | | 0.0421 |
| ENSG00000166710 | B2M | 1085.38 | | 853.90 | | −0.35 | | 0.04225 |
| ENSG00000197816 | CCDC180 | 1.39 | | 0.55 | | −1.35 | | 0.0427 |
| ENSG00000127184 | COX7C | 326.74 | | 256.73 | | −0.35 | | 0.0429 |
| ENSG00000170142 | UBE2E1 | 175.28 | | 136.02 | | −0.37 | | 0.04295 |
| ENSG00000146223 | RPL7L1 | 107.71 | | 79.79 | | −0.43 | | 0.0432 |
| ENSG00000078668 | VDAC3 | 107.69 | | 84.24 | | −0.35 | | 0.04335 |
| ENSG00000070669 | ASNS | 25.19 | | 32.97 | | 0.39 | | 0.0436 |
| ENSG00000167863 | ATP5H | 173.90 | | 133.12 | | −0.39 | | 0.04365 |
| ENSG00000168676 | KCTD19 | 0.33 | | 1.13 | | 1.79 | | 0.04375 |
| ENSG00000221955 | SLC12A8 | 15.51 | | 11.65 | | −0.41 | | 0.0438 |
| ENSG00000170891 | CYTL1 | 6.30 | | 4.12 | | −0.61 | | 0.0439 |
| ENSG00000159593 | NAE1 | 85.40 | | 67.66 | | −0.34 | | 0.0439 |
| ENSG00000166851 | PLK1 | 16.28 | | 24.26 | | 0.58 | | 0.04405 |
| ENSG00000131037 | EPS8L1 | 1.77 | | 0.99 | | −0.84 | | 0.0442 |
| ENSG00000239776 | AC079949.1 | 0.00 | | 1.46 | | inf | | 0.04445 |
| ENSG00000187605 | TET3 | 3.16 | | 4.22 | | 0.42 | | 0.04465 |
| ENSG00000204706 | MAMDC2−AS1 | 1.22 | | 2.33 | | 0.94 | | 0.0447 |
| ENSG00000100814 | CCNB1IP1 | 46.36 | | 34.92 | | −0.41 | | 0.04475 |
| ENSG00000130699 | TAF4 | 2.45 | | 3.57 | | 0.54 | | 0.045 |
| ENSG00000109846 | CRYAB | 19.74 | | 28.23 | | 0.52 | | 0.04525 |
| ENSG00000114391 | RPL24 | 963.03 | | 767.95 | | −0.33 | | 0.0453 |
| ENSG00000184012 | TMPRSS2 | 14.30 | | 9.17 | | −0.64 | | 0.0454 |
| ENSG00000125821 | DTD1 | 52.43 | | 37.34 | | −0.49 | | 0.0454 |
| ENSG00000198416 | ZNF658B | 1.30 | | 0.81 | | −0.68 | | 0.0459 |
| ENSG00000164548 | TRA2A | 57.92 | | 79.36 | | 0.45 | | 0.04595 |
| ENSG00000134419 | RPS15A | 1491.86 | | 1108.12 | | −0.43 | | 0.04605 |
| ENSG00000238266 | LINC00707 | 0.97 | | 1.55 | | 0.68 | | 0.04615 |
| ENSG00000168488 | ATXN2L | 13.95 | | 20.98 | | 0.59 | | 0.04665 |
| ENSG00000140836 | ZFHX3 | 2.09 | | 2.96 | | 0.50 | | 0.04785 |
| ENSG00000141524 | TMC6 | 1.14 | | 0.57 | | −1.00 | | 0.0482 |
| ENSG00000101052 | IFT52 | 25.59 | | 18.19 | | −0.49 | | 0.04825 |
| ENSG00000186446 | ZNF501 | 3.09 | | 1.96 | | −0.66 | | 0.0484 |
| ENSG00000130158 | DOCK6 | 0.75 | | 1.46 | | 0.96 | | 0.04845 |
| ENSG00000153982 | GDPD1 | 4.97 | | 3.11 | | −0.67 | | 0.04865 |
| ENSG00000278771 | Metazoa_SRP | 206.42 | | 157.17 | | −0.39 | | 0.0487 |
| ENSG00000130766 | SESN2 | 1.98 | | 2.88 | | 0.54 | | 0.0491 |
| ENSG00000126107 | HECTD3 | 3.33 | | 4.98 | | 0.58 | | 0.04935 |
| ENSG00000148803 | FUOM | 3.66 | | 6.06 | | 0.73 | | 0.04935 |
| ENSG00000110080 | ST3GAL4 | 6.69 | | 4.44 | | −0.59 | | 0.04975 |
| ENSG00000071082 | RPL31 | 708.14 | | 545.05 | | −0.38 | | 0.04995 |
| ENSG00000111144 | LTA4H | 78.35 | | 62.42 | | −0.33 | | 0.04995 |

| **ReNcell CX 72 h** | | |  |  |  |  |
| --- | --- | --- | --- | --- | --- | --- |
| **Gene ID** | **Gene** | **Value_1** | | **Value_2** | **log2 (Fold change)** | ***p* value** |
| ENSG00000065609 | SNAP91 | 1.13 | | 0.33 | −1.80 | 0.00035 |
| ENSG00000166922 | SCG5 | 1.73 | | 0.43 | −2.01 | 0.0007 |
| ENSG00000176978 | DPP7 | 8.11 | | 17.24 | 1.09 | 0.00095 |
| ENSG00000171246 | NPTX1 | 3.20 | | 1.69 | −0.92 | 0.0012 |
| ENSG00000106683 | LIMK1 | 17.31 | | 26.49 | 0.61 | 0.0012 |
| ENSG00000187840 | EIF4EBP1 | 35.03 | | 53.44 | 0.61 | 0.0013 |
| ENSG00000281508 | CDR1−AS | 2.35 | | 0.49 | −2.25 | 0.0015 |
| ENSG00000255823 | MTRNR2L8 | 16.19 | | 8.37 | −0.95 | 0.00205 |
| ENSG00000148677 | ANKRD1 | 11.25 | | 16.93 | 0.59 | 0.0023 |
| ENSG00000229956 | ZRANB2−AS2 | 1.06 | | 0.52 | −1.02 | 0.0032 |
| ENSG00000119681 | LTBP2 | 14.36 | | 19.70 | 0.46 | 0.0032 |
| ENSG00000173801 | JUP | 4.11 | | 6.32 | 0.62 | 0.00365 |
| ENSG00000256618 | MTRNR2L1 | 15.63 | | 8.30 | −0.91 | 0.0039 |
| ENSG00000134121 | CHL1 | 2.04 | | 0.56 | −1.87 | 0.0042 |
| ENSG00000112245 | PTP4A1 | 58.93 | | 40.92 | −0.53 | 0.00435 |
| ENSG00000198892 | SHISA4 | 8.66 | | 13.88 | 0.68 | 0.00575 |
| ENSG00000122490 | PQLC1 | 2.09 | | 3.95 | 0.91 | 0.00595 |
| ENSG00000130731 | C16orf13 | 17.26 | | 26.94 | 0.64 | 0.00605 |
| ENSG00000123560 | PLP1 | 4.82 | | 7.18 | 0.57 | 0.00635 |
| ENSG00000179915 | NRXN1 | 1.07 | | 0.45 | −1.26 | 0.008 |
| ENSG00000101057 | MYBL2 | 4.36 | | 6.68 | 0.62 | 0.008 |
| ENSG00000049540 | ELN | 5.04 | | 7.40 | 0.55 | 0.00805 |
| ENSG00000198739 | LRRTM3 | 1.09 | | 0.30 | −1.85 | 0.00825 |
| ENSG00000181722 | ZBTB20 | 10.14 | | 16.96 | 0.74 | 0.009 |
| ENSG00000180638 | SLC47A2 | 1.12 | | 0.56 | −0.99 | 0.01055 |
| ENSG00000105185 | PDCD5 | 273.68 | | 205.81 | −0.41 | 0.01075 |
| ENSG00000136717 | BIN1 | 8.39 | | 12.55 | 0.58 | 0.0118 |
| ENSG00000165886 | UBTD1 | 3.13 | | 5.07 | 0.69 | 0.01245 |
| ENSG00000182809 | CRIP2 | 4.30 | | 7.49 | 0.80 | 0.01315 |
| ENSG00000123159 | GIPC1 | 14.17 | | 21.32 | 0.59 | 0.01385 |
| ENSG00000063245 | EPN1 | 3.00 | | 4.84 | 0.69 | 0.0145 |
| ENSG00000121897 | LIAS | 14.21 | | 9.26 | −0.62 | 0.01565 |
| ENSG00000128283 | CDC42EP1 | 1.56 | | 3.03 | 0.96 | 0.01565 |
| ENSG00000162066 | AMDHD2 | 0.69 | | 1.84 | 1.41 | 0.0157 |
| ENSG00000175573 | C11orf68 | 7.62 | | 11.02 | 0.53 | 0.0159 |
| ENSG00000079999 | KEAP1 | 19.62 | | 29.10 | 0.57 | 0.0164 |
| ENSG00000102554 | KLF5 | 8.97 | | 14.06 | 0.65 | 0.0164 |
| ENSG00000141985 | SH3GL1 | 10.67 | | 15.93 | 0.58 | 0.0165 |
| ENSG00000162576 | MXRA8 | 15.63 | | 21.85 | 0.48 | 0.01665 |
| ENSG00000143409 | FAM63A | 2.39 | | 3.90 | 0.71 | 0.01725 |
| ENSG00000173812 | EIF1 | 85.67 | | 62.80 | −0.45 | 0.01795 |
| ENSG00000262246 | CORO7 | 1.75 | | 3.11 | 0.83 | 0.018 |
| ENSG00000198792 | TMEM184B | 9.78 | | 17.10 | 0.81 | 0.0187 |
| ENSG00000247596 | TWF2 | 10.62 | | 16.60 | 0.64 | 0.0188 |
| ENSG00000131126 | TEX101 | 5.29 | | 2.90 | −0.87 | 0.0193 |
| ENSG00000163517 | HDAC11 | 1.32 | | 2.40 | 0.86 | 0.01975 |
| ENSG00000125817 | CENPB | 8.60 | | 11.65 | 0.44 | 0.0199 |
| ENSG00000175756 | AURKAIP1 | 8.61 | | 12.87 | 0.58 | 0.0202 |
| ENSG00000181135 | ZNF707 | 0.91 | | 1.65 | 0.85 | 0.02025 |
| ENSG00000130055 | GDPD2 | 0.83 | | 1.55 | 0.91 | 0.02055 |
| ENSG00000222328 | RNU2−2P | 435.98 | | 711.38 | 0.71 | 0.02085 |
| ENSG00000163346 | PBXIP1 | 16.20 | | 21.20 | 0.39 | 0.0212 |
| ENSG00000076706 | MCAM | 23.35 | | 29.83 | 0.35 | 0.0213 |
| ENSG00000106211 | HSPB1 | 16.01 | | 24.37 | 0.61 | 0.0213 |
| ENSG00000074219 | TEAD2 | 24.69 | | 35.48 | 0.52 | 0.0218 |
| ENSG00000135916 | ITM2C | 18.64 | | 27.49 | 0.56 | 0.0222 |
| ENSG00000042493 | CAPG | 20.27 | | 29.52 | 0.54 | 0.02265 |
| ENSG00000142871 | CYR61 | 27.03 | | 35.48 | 0.39 | 0.0229 |
| ENSG00000199347 | RNU5E−1 | 14.08 | | 26.88 | 0.93 | 0.0236 |
| ENSG00000106404 | CLDN15 | 0.72 | | 1.33 | 0.88 | 0.02455 |
| ENSG00000258943 | RP11−696D21.2 | 0.36 | | 1.18 | 1.73 | 0.02455 |
| ENSG00000107796 | ACTA2 | 232.41 | | 300.68 | 0.37 | 0.02545 |
| ENSG00000123977 | DAW1 | 2.94 | | 1.72 | −0.77 | 0.02555 |
| ENSG00000272341 | RP1−151F17.2 | 2.55 | | 1.59 | −0.68 | 0.02555 |
| ENSG00000142089 | IFITM3 | 15.48 | | 22.01 | 0.51 | 0.02575 |
| ENSG00000178896 | EXOSC4 | 6.00 | | 9.36 | 0.64 | 0.02615 |
| ENSG00000197837 | HIST4H4 | 1.89 | | 3.77 | 0.99 | 0.02615 |
| ENSG00000159363 | ATP13A2 | 28.58 | | 41.95 | 0.55 | 0.0263 |
| ENSG00000163702 | IL17RC | 3.59 | | 5.77 | 0.69 | 0.02655 |
| ENSG00000130165 | ELOF1 | 22.91 | | 31.24 | 0.45 | 0.02685 |
| ENSG00000214111 | RP1−258N20.3 | 0.93 | | 2.20 | 1.24 | 0.02685 |
| ENSG00000167967 | E4F1 | 1.43 | | 2.38 | 0.73 | 0.02705 |
| ENSG00000167797 | CDK2AP2 | 3.52 | | 5.88 | 0.74 | 0.0274 |
| ENSG00000104894 | CD37 | 0.50 | | 1.17 | 1.22 | 0.02765 |
| ENSG00000127445 | PIN1 | 22.41 | | 31.59 | 0.50 | 0.0278 |
| ENSG00000110723 | EXPH5 | 3.19 | | 2.13 | −0.58 | 0.02795 |
| ENSG00000092529 | CAPN3 | 0.44 | | 1.03 | 1.22 | 0.0283 |
| ENSG00000178685 | PARP10 | 1.31 | | 2.73 | 1.06 | 0.0285 |
| ENSG00000131591 | C1orf159 | 1.09 | | 1.98 | 0.87 | 0.02875 |
| ENSG00000171130 | ATP6V0E2 | 37.03 | | 47.58 | 0.36 | 0.0289 |
| ENSG00000204542 | C6orf15 | 8.36 | | 12.00 | 0.52 | 0.02905 |
| ENSG00000166002 | SMCO4 | 0.99 | | 2.34 | 1.24 | 0.0293 |
| ENSG00000100304 | TTLL12 | 15.91 | | 23.09 | 0.54 | 0.02945 |
| ENSG00000179922 | ZNF784 | 1.22 | | 2.07 | 0.76 | 0.02975 |
| ENSG00000061337 | LZTS1 | 8.13 | | 10.54 | 0.37 | 0.02985 |
| ENSG00000188596 | CFAP54 | 0.83 | | 1.53 | 0.88 | 0.03 |
| ENSG00000175221 | MED16 | 2.28 | | 3.96 | 0.80 | 0.0303 |
| ENSG00000163584 | RPL22L1 | 229.25 | | 177.38 | −0.37 | 0.0313 |
| ENSG00000184524 | CEND1 | 0.75 | | 1.47 | 0.97 | 0.0313 |
| ENSG00000167767 | KRT80 | 4.92 | | 6.64 | 0.43 | 0.0315 |
| ENSG00000185742 | C11orf87 | 3.17 | | 2.30 | −0.46 | 0.0321 |
| ENSG00000154027 | AK5 | 1.90 | | 0.83 | −1.19 | 0.03225 |
| ENSG00000255633 | MTRNR2L9 | 1.11 | | 0.00 | #NAME? | 0.03255 |
| ENSG00000278748 | AC021037.2 | 1.37 | | 0.00 | #NAME? | 0.03255 |
| ENSG00000123143 | PKN1 | 12.97 | | 18.70 | 0.53 | 0.0326 |
| ENSG00000104435 | STMN2 | 2.67 | | 1.26 | −1.08 | 0.03285 |
| ENSG00000114812 | VIPR1 | 1.23 | | 2.06 | 0.75 | 0.03305 |
| ENSG00000239467 | AC007405.6 | 2.10 | | 0.93 | −1.17 | 0.03325 |
| ENSG00000115268 | RPS15 | 151.82 | | 193.02 | 0.35 | 0.0339 |
| ENSG00000183087 | GAS6 | 1.94 | | 2.97 | 0.62 | 0.034 |
| ENSG00000179862 | CITED4 | 0.51 | | 1.13 | 1.14 | 0.03405 |
| ENSG00000235173 | HGH1 | 2.43 | | 3.71 | 0.61 | 0.03455 |
| ENSG00000163485 | ADORA1 | 5.01 | | 7.67 | 0.61 | 0.0349 |
| ENSG00000249072 | RP11−777B9.5 | 1.88 | | 0.06 | −4.90 | 0.035 |
| ENSG00000067221 | STOML1 | 1.01 | | 1.93 | 0.93 | 0.0351 |
| ENSG00000270011 | ZNF559−ZNF177 | 1.03 | | 2.69 | 1.38 | 0.0351 |
| ENSG00000168899 | VAMP5 | 10.17 | | 14.75 | 0.54 | 0.03555 |
| ENSG00000138172 | CALHM2 | 1.42 | | 2.66 | 0.90 | 0.03565 |
| ENSG00000232445 | RP11−132A1.4 | 2.99 | | 1.50 | −0.99 | 0.0361 |
| ENSG00000104859 | CLASRP | 13.17 | | 19.62 | 0.57 | 0.03615 |
| ENSG00000108846 | ABCC3 | 9.85 | | 14.38 | 0.55 | 0.03625 |
| ENSG00000135480 | KRT7 | 5.02 | | 8.09 | 0.69 | 0.0363 |
| ENSG00000269028 | MTRNR2L12 | 39.62 | | 25.58 | −0.63 | 0.0364 |
| ENSG00000196923 | PDLIM7 | 11.91 | | 16.29 | 0.45 | 0.0364 |
| ENSG00000208037 | MIR320A | 2.57 | | 9.95 | 1.95 | 0.03645 |
| ENSG00000014216 | CAPN1 | 12.91 | | 20.64 | 0.68 | 0.03675 |
| ENSG00000150995 | ITPR1 | 5.07 | | 3.66 | −0.47 | 0.037 |
| ENSG00000128185 | DGCR6L | 1.72 | | 3.10 | 0.85 | 0.03815 |
| ENSG00000236992 | RPL12L3 | 3.79 | | 1.80 | −1.07 | 0.03825 |
| ENSG00000275713 | HIST1H2BH | 84.88 | | 109.98 | 0.37 | 0.0385 |
| ENSG00000269893 | SNHG8 | 70.51 | | 51.83 | −0.44 | 0.03855 |
| ENSG00000138495 | COX17 | 31.65 | | 22.73 | −0.48 | 0.0388 |
| ENSG00000127054 | CPSF3L | 30.32 | | 45.06 | 0.57 | 0.03905 |
| ENSG00000197409 | HIST1H3D | 8.67 | | 13.61 | 0.65 | 0.03915 |
| ENSG00000179115 | FARSA | 21.45 | | 29.13 | 0.44 | 0.03945 |
| ENSG00000159840 | ZYX | 27.13 | | 39.87 | 0.56 | 0.03945 |
| ENSG00000111911 | HINT3 | 13.35 | | 10.40 | −0.36 | 0.03965 |
| ENSG00000127184 | COX7C | 320.68 | | 254.89 | −0.33 | 0.04015 |
| ENSG00000108691 | CCL2 | 250.79 | | 319.79 | 0.35 | 0.04015 |
| ENSG00000114923 | SLC4A3 | 1.08 | | 1.72 | 0.67 | 0.0405 |
| ENSG00000169710 | FASN | 13.15 | | 16.73 | 0.35 | 0.0407 |
| ENSG00000198918 | RPL39 | 959.02 | | 765.68 | −0.32 | 0.04075 |
| ENSG00000156463 | SH3RF2 | 0.56 | | 1.26 | 1.16 | 0.04155 |
| ENSG00000160326 | SLC2A6 | 2.32 | | 3.42 | 0.56 | 0.04175 |
| ENSG00000064666 | CNN2 | 52.04 | | 70.18 | 0.43 | 0.04195 |
| ENSG00000162734 | PEA15 | 109.43 | | 142.32 | 0.38 | 0.0425 |
| ENSG00000110958 | PTGES3 | 108.08 | | 86.95 | −0.31 | 0.04295 |
| ENSG00000116133 | DHCR24 | 84.04 | | 105.18 | 0.32 | 0.04325 |
| ENSG00000142546 | NOSIP | 15.49 | | 22.69 | 0.55 | 0.044 |
| ENSG00000130479 | MAP1S | 2.50 | | 4.16 | 0.74 | 0.0451 |
| ENSG00000221914 | PPP2R2A | 36.53 | | 27.74 | −0.40 | 0.0456 |
| ENSG00000145012 | LPP | 25.90 | | 35.28 | 0.45 | 0.0462 |
| ENSG00000183580 | FBXL7 | 2.19 | | 1.50 | −0.55 | 0.0463 |
| ENSG00000261236 | BOP1 | 6.98 | | 9.91 | 0.51 | 0.04645 |
| ENSG00000221676 | RNU6ATAC | 1.28 | | 6.30 | 2.30 | 0.0469 |
| ENSG00000166165 | CKB | 126.98 | | 164.04 | 0.37 | 0.04705 |
| ENSG00000072080 | SPP2 | 2.21 | | 0.36 | −2.62 | 0.04715 |
| ENSG00000091428 | RAPGEF4 | 2.31 | | 1.20 | −0.94 | 0.04735 |
| ENSG00000128284 | APOL3 | 1.17 | | 0.65 | −0.86 | 0.04755 |
| ENSG00000163347 | CLDN1 | 2.72 | | 3.78 | 0.47 | 0.04785 |
| ENSG00000188243 | COMMD6 | 194.16 | | 154.91 | −0.33 | 0.04795 |
| ENSG00000173465 | SSSCA1 | 3.53 | | 6.50 | 0.88 | 0.048 |
| ENSG00000111667 | USP5 | 30.98 | | 43.77 | 0.50 | 0.0482 |
| ENSG00000235217 | TSPY26P | 1.53 | | 2.20 | 0.52 | 0.04825 |
| ENSG00000141040 | ZNF287 | 6.96 | | 3.98 | −0.81 | 0.0483 |
| ENSG00000170454 | KRT75 | 1.54 | | 2.41 | 0.64 | 0.0487 |
| ENSG00000167693 | NXN | 20.53 | | 28.98 | 0.50 | 0.04885 |
| ENSG00000182004 | SNRPE | 144.93 | | 112.88 | −0.36 | 0.04895 |
| ENSG00000177352 | CCDC71 | 3.43 | | 4.94 | 0.53 | 0.049 |
| ENSG00000166896 | XRCC6BP1 | 6.28 | | 3.98 | −0.66 | 0.0492 |
| ENSG00000198795 | ZNF521 | 3.96 | | 6.27 | 0.66 | 0.04945 |
| ENSG00000279519 | RP11−288C18.1 | 1.35 | | 0.81 | −0.74 | 0.0497 |
| ENSG00000162688 | AGL | 8.25 | | 5.79 | −0.51 | 0.0497 |
| ENSG00000133027 | PEMT | 3.76 | | 5.86 | 0.64 | 0.0498 |

**Table S4**. Gene Networks Differentially Expressed Following *MSNP1AS* Over−Expression.

| **SK-N-SH 24 hours** |  |  |  |
| --- | --- | --- | --- |
| **Term** | **PValue** | **Fold Enrichment** | **Bonferroni** |
| GO:0010033~response to organic substance | 9.27E−05 | 3.10 | 1.02E−01 |
| GO:0006915~apoptosis | 1.64E−04 | 3.27 | 1.73E−01 |
| GO:0012501~programmed cell death | 1.91E−04 | 3.22 | 1.99E−01 |
| GO:0007167~enzyme linked receptor protein signaling pathway | 2.53E−04 | 4.22 | 2.55E−01 |
| GO:0008219~cell death | 3.07E−04 | 2.92 | 3.00E−01 |
| GO:0016265~death | 3.30E−04 | 2.90 | 3.19E−01 |
| GO:0044421~extracellular region part | 4.99E−04 | 2.55 | 9.14E−02 |
| GO:0001568~blood vessel development | 5.23E−04 | 4.82 | 4.56E−01 |
| GO:0042060~wound healing | 5.98E−04 | 5.50 | 5.01E−01 |
| GO:0001944~vasculature development | 6.14E−04 | 4.71 | 5.10E−01 |
| GO:0050878~regulation of body fluid levels | 6.83E−04 | 6.52 | 5.48E−01 |
| GO:0005576~extracellular region | 8.23E−04 | 1.89 | 1.46E−01 |
| GO:0009719~response to endogenous stimulus | 9.49E−04 | 3.57 | 6.68E−01 |

| **SK-N-SH 72 h** |  |  |  |
| --- | --- | --- | --- |
| **Term** | ***p* Value** | **Fold Enrichment** | **Bonferroni** |
| GO:0006414~translational elongation | 2.68E−14 | 10.63 | 4.72E−11 |
| GO:0022626~cytosolic ribosome | 1.29E−13 | 11.69 | 3.87E−11 |
| GO:0033279~ribosomal subunit | 3.09E−11 | 7.81 | 9.31E−09 |
| GO:0003735~structural constituent of ribosome | 2.36E−10 | 6.47 | 1.15E−07 |
| GO:0044445~cytosolic part | 5.60E−10 | 6.58 | 1.69E−07 |
| GO:0034728~nucleosome organization | 1.61E−09 | 8.66 | 2.83E−06 |
| GO:0000786~nucleosome | 1.84E−09 | 10.85 | 5.52E−07 |
| GO:0005840~ribosome | 4.45E−09 | 5.14 | 1.34E−06 |
| GO:0032993~protein−DNA complex | 7.44E−09 | 8.56 | 2.24E−06 |
| GO:0006323~DNA packaging | 3.35E−08 | 6.88 | 5.91E−05 |
| GO:0022627~cytosolic small ribosomal subunit | 4.42E−08 | 13.15 | 1.33E−05 |
| GO:0006334~nucleosome assembly | 4.43E−08 | 8.31 | 7.81E−05 |
| GO:0006412~translation | 5.51E−08 | 3.89 | 9.72E−05 |
| GO:0031497~chromatin assembly | 6.62E−08 | 8.02 | 1.17E−04 |
| GO:0065004~protein−DNA complex assembly | 1.10E−07 | 7.67 | 1.94E−04 |
| GO:0006333~chromatin assembly or disassembly | 6.59E−07 | 5.92 | 1.16E−03 |
| GO:0015935~small ribosomal subunit | 2.62E−06 | 8.35 | 7.87E−04 |
| GO:0005829~cytosol | 3.45E−06 | 1.98 | 1.04E−03 |
| GO:0006325~chromatin organization | 7.78E−06 | 3.12 | 1.36E−02 |
| GO:0051276~chromosome organization | 1.21E−05 | 2.77 | 2.12E−02 |
| GO:0015934~large ribosomal subunit | 3.66E−05 | 7.07 | 1.10E−02 |
| GO:0034621~cellular macromolecular complex subunit organization | 3.83E−05 | 3.01 | 6.54E−02 |
| GO:0022625~cytosolic large ribosomal subunit | 7.19E−05 | 9.69 | 2.14E−02 |
| GO:0034622~cellular macromolecular complex assembly | 9.33E−05 | 3.04 | 1.52E−01 |
| GO:0005198~structural molecule activity | 1.02E−04 | 2.31 | 4.89E−02 |
| GO:0000785~chromatin | 1.12E−04 | 3.68 | 3.32E−02 |
| GO:0030529~ribonucleoprotein complex | 1.21E−04 | 2.45 | 3.56E−02 |
| GO:0065003~macromolecular complex assembly | 6.30E−04 | 2.10 | 6.71E−01 |
| GO:0043933~macromolecular complex subunit organization | 7.23E−04 | 2.04 | 7.21E−01 |

| **ReNcell CX 24 h** |  |  |  |
| --- | --- | --- | --- |
| **Term** | ***p* Value** | **Fold Enrichment** | **Bonferroni** |
| GO:0006414~translational elongation | 2.14E−26 | 19.44 | 3.33E−23 |
| GO:0003735~structural constituent of ribosome | 1.97E−22 | 12.18 | 7.27E−20 |
| GO:0022626~cytosolic ribosome | 3.47E−22 | 21.04 | 9.29E−20 |
| GO:0005840~ribosome | 1.35E−20 | 10.45 | 3.62E−18 |
| GO:0033279~ribosomal subunit | 2.67E−20 | 14.53 | 7.15E−18 |
| GO:0006412~translation | 1.17E−16 | 6.81 | 1.72E−13 |
| GO:0044445~cytosolic part | 3.44E−16 | 11.21 | 8.93E−14 |
| GO:0022627~cytosolic small ribosomal subunit | 5.44E−14 | 25.18 | 1.46E−11 |
| GO:0030529~ribonucleoprotein complex | 2.53E−12 | 4.66 | 6.79E−10 |
| GO:0005198~structural molecule activity | 2.54E−12 | 4.01 | 9.39E−10 |
| GO:0005829~cytosol | 3.73E−12 | 2.91 | 9.99E−10 |
| GO:0015935~small ribosomal subunit | 2.01E−11 | 15.99 | 5.39E−09 |
| GO:0015934~large ribosomal subunit | 1.21E−08 | 12.72 | 3.25E−06 |
| GO:0022625~cytosolic large ribosomal subunit | 2.18E−08 | 18.35 | 5.85E−06 |
| GO:0003723~RNA binding | 2.63E−07 | 2.95 | 9.72E−05 |
| GO:0043228~non−membrane−bounded organelle | 9.08E−07 | 1.82 | 2.43E−04 |
| GO:0043232~intracellular non−membrane−bounded organelle | 9.08E−07 | 1.82 | 2.43E−04 |
| GO:0042981~regulation of apoptosis | 9.89E−05 | 2.35 | 1.42E−01 |
| GO:0043067~regulation of programmed cell death | 1.16E−04 | 2.33 | 1.64E−01 |
| GO:0010941~regulation of cell death | 1.23E−04 | 2.32 | 1.73E−01 |
| GO:0016563~transcription activator activity | 2.27E−04 | 2.93 | 8.04E−02 |
| GO:0000123~histone acetyltransferase complex | 4.35E−04 | 9.30 | 1.10E−01 |
| GO:0003712~transcription cofactor activity | 6.31E−04 | 2.92 | 2.08E−01 |
| GO:0051726~regulation of cell cycle | 6.37E−04 | 3.08 | 6.28E−01 |
| GO:0010604~positive regulation of macromolecule metabolic process | 6.40E−04 | 2.12 | 6.30E−01 |
| GO:0043065~positive regulation of apoptosis | 8.40E−04 | 2.71 | 7.29E−01 |
| GO:0043068~positive regulation of programmed cell death | 9.01E−04 | 2.69 | 7.53E−01 |
| GO:0003713~transcription coactivator activity | 9.14E−04 | 3.63 | 2.86E−01 |
| GO:0010942~positive regulation of cell death | 9.44E−04 | 2.68 | 7.69E−01 |
| GO:0042542~response to hydrogen peroxide | 9.84E−04 | 7.79 | 7.83E−01 |

| **ReNcell CX 72 h** |  |  |  |
| --- | --- | --- | --- |
| **Term** | ***p* Value** | **Fold Enrichment** | **Bonferroni** |
| GO:0043232~intracellular non−membrane−bounded organelle | 2.05E−03 | 1.63 | 3.46E−01 |
| GO:0043228~non−membrane−bounded organelle | 2.05E−03 | 1.63 | 3.46E−01 |
| GO:0005856~cytoskeleton | 2.43E−03 | 1.95 | 3.96E−01 |
| GO:0032268~regulation of cellular protein metabolic process | 4.58E−03 | 2.88 | 9.84E−01 |
| GO:0044430~cytoskeletal part | 1.38E−02 | 1.97 | 9.44E−01 |
| GO:0044456~synapse part | 1.80E−02 | 3.34 | 9.76E−01 |
| GO:0050840~extracellular matrix binding | 1.89E−02 | 14.01 | 9.97E−01 |
| GO:0005911~cell−cell junction | 2.24E−02 | 3.70 | 9.91E−01 |
| GO:0042734~presynaptic membrane | 2.48E−02 | 12.13 | 9.94E−01 |
| GO:0005198~structural molecule activity | 2.71E−02 | 2.19 | 1.00E+00 |
| GO:0005829~cytosol | 2.95E−02 | 1.68 | 9.98E−01 |
| GO:0045202~synapse | 3.08E−02 | 2.64 | 9.98E−01 |
